# Vaccination potential of B and T epitope-enriched NP and M2 against Influenza A viruses from different clades and hosts

**DOI:** 10.1371/journal.pone.0191574

**Published:** 2018-01-29

**Authors:** Irina Tutykhina, Ilias Esmagambetov, Alexander Bagaev, Alexey Pichugin, Andrey Lysenko, Dmitry Shcherbinin, Elena Sedova, Denis Logunov, Maxim Shmarov, Ravshan Ataullakhanov, Boris Naroditsky, Alexander Gintsburg

**Affiliations:** 1 Federal Research Centre of Epidemiology and Microbiology named after Honorary Academician N. F. Gamaleya, Ministry of Health, Moscow, Russia; 2 The Institute of Immunology, Federal Medical-Biological Agency, Moscow, Russia; Monash University, AUSTRALIA

## Abstract

To avoid outbreaks of influenza virus epidemics and pandemics among human populations, modern medicine requires the development of new universal vaccines that are able to provide protection from a wide range of influenza A virus strains. In the course of development of a universal vaccine, it is necessary to consider that immunity must be generated even against viruses from different hosts because new human epidemic virus strains have their origins in viruses of birds and other animals. We have enriched conserved viral proteins–nucleoprotein (NP) and matrix protein 2 (M2)—by B and T-cell epitopes not only human origin but also swine and avian origin. For this purpose, we analyzed M2 and NP sequences with respect to changes in the sequences of known T and B-cell epitopes and chose conserved and evolutionarily significant epitopes. Eventually, we found consensus sequences of M2 and NP that have the maximum quantity of epitopes that are 100% coincident with them. Consensus epitope-enriched amino acid sequences of M2 and NP proteins were included in a recombinant adenoviral vector. Immunization with Ad5-tet-M2NP induced strong CD8 and CD4 T cells responses, specific to each of the encoded antigens, i.e. M2 and NP. Eight months after immunization with Ad5-tet-M2NP, high numbers of M2- and NP-responding “effector memory” CD44posCD62neg T cells were found in the mouse spleens, which revealed a long-term T cell immune memory conferred by the immunization. In all, the challenge experiments showed an extraordinarily wide-ranging efficacy of protection by the Ad5-tet-M2NP vaccine, covering 5 different heterosubtypes of influenza A virus (2 human, 2 avian and 1 swine).

## Introduction

Influenza viral disease remains one of the most significant global challenges. Half a million annual deaths and globally spread morbidity in humans are caused by seasonal epidemic strains of influenza viruses. Moreover, potentially pandemic strains, such as so-called “bird flu” H5N1 and “swine flu” H1N1, are a constant threat because of their significant mortality rates (greater than 50% for H5N1) [1–4].

Currently licensed influenza vaccines target seasonal virus strains and must be promptly changed every 1 or 2 years. A universal influenza vaccine that would effectively protect from any seasonally appearing as well as potential pandemic strains is considered to be an ultimate research goal [5]. The mechanism of action of a “universal” vaccine depends on the induction of broad-spectrum immune responses, e.g., the occurrence of heterosubtypic immunity in an organism. Under these circumstances, virus replication and transmission should be restricted, consequently lowering morbidity and mortality caused by influenza.

Immune responses to influenza virus are multifactorial, and antibodies, B-cells, CD8+ and CD4+ T-cells are all essential for effective viral clearance and to prevent reinfection. [6]. To avoid neutralization by the immune system, a common strategy of influenza virus is mutational variability of all its proteins. In addition to mutations of surface proteins, which serves to avoid virus neutralization by antibodies, mutations generated in more conserved inner proteins serve to avoid exposure to CTL-mediated immunity. Despite this, the development of a universal vaccine is theoretically possible because several antigens have epitopes that are conserved, even among distant virus strains. It has been suggested that one of the reasons for the emergence of conserved B-cell epitopes is a deficiency or low-level affinity of antibodies to this site, and its subsequent inaccessibility to selective pressures. Such epitopes typically are in the “stalk” domain of hemagglutinin (HA) and the ectodomain of matrix 2 protein (M2e). [7,8]. A series of experiments have shown that in vitro passaging of influenza virus with high-affinity antibodies against the “stalk” domain of hemagglutinin leads to the emergence of escape-mutants within only a few passages. [9]. The same effect is also observed in vitro with use of antibodies against M2e. [10]. These data allow us to suggest that vaccines whose effect is based on antibodies against conserved influenza virus epitopes, may become ineffective due to the possible emergence of escape-mutants in the population. At the same time, it seems that antibodies are still necessary for elimination of the virus, as shown in the example of animal immunization with preparations using NP as an antigen. [11].

According to many animal and human studies, heterosubtypic immunity against influenza virus is T-cell-based via conserved T cell epitopes. The emergence of conserved T-cell epitopes has been suggested to develop from the fact that any changes in sequences of these epitopes leads to loss of functions of whole proteins. However, few such epitopes have been found to date. Moreover, not all humans are potentially able to form immune responses to one or more T-cell epitopes [12]. The majority of conserved T-cell epitopes are found in the inner proteins of influenza virus (M1, NP, PA, PB1) [13]. There are many reports in the literature that vaccines based on these proteins are heterosubtypic, and they are furthermore able to protect against viruses isolated from different hosts. For example, it is known that a vaccine based on the M2 protein ectodomain consensus sequence protects animals from different strains of influenza virus that originate from humans and birds [14]. The animal studies from Epstein et al and Price et al used a mixture of adenoviral vectors that expressed full-length M2 and nucleoprotein [15, 16]. These experiments showed that such immunization leads to antibody production, induction of T-cell responses and protection from influenza virus heterosubtypes. Moreover, a combination of these components in the vaccine lowers the level of influenza virus transmission.

We have suggested that the enrichment of conserved viral proteins by B and T-cell epitopes may become one of the strategies to develop a universal vaccine against influenza virus. However, there are also other similar strategies [17, 18]. In the course of development of a universal vaccine, it is necessary to consider that immunity must be generated even against viruses from different hosts because new human epidemic virus strains have their origins in viruses of birds and other animals [19]. Therefore, it is essential to create sequences of proteins that contain both human and animal (i.e., bird and swine) T and B-cell epitopes.

To obtain a preparation that provides effective immunization against a wide range of different strains of influenza A virus from different hosts, in our work we have chosen the M2 protein and NP protein as target antigens. The M2 antigen has been chosen as a component that stimulates general humoral immune responses; the NP antigen as a component that stimulates T-cell immune responses.

Sequences of M2 and NP proteins of influenza A virus that have circulated among human beings for the last 100 years have been analyzed with regards to changes in speciation of B and T-cell epitopes that are currently known. Consensus sequences from these proteins were modified to increase the content of not only human, but of swine and bird influenza virus epitopes as well. The generated sequences of M2 and NP proteins enriched with B and T-cell epitopes were included in an adenovirus vector. We show that a single intranasal administration of Ad-tet-M2NP induces strong systemic antibody and T-cell responses, and confers an extraordinary heterosubtypic protection from lethal challenge with 5 influenza virus strains belonging to 3 different clades (clade H1—H1N1 (A/USSR/90/77), H2N3 (A/BlackDuck/NewJersey/1580/78), and H5N2 (A/Duck(Mallard)/Pennsylvania/10218/84); clade H3—H3N2 (A/Aichi/2/68); H9—H9N2 (A/Swine/Hong Kong/9A-1/98)) and 3 different original hosts (Homo sapiens, Gallus gallus and Sus domesticus). One month post-immunization, mice produced high levels of serum antibodies capable of binding synthetic M2 and recombinant NP proteins, as well as lysates of the mentioned above influenza viruses. Strong IFN-γ-secreting T-cell responses to each of the listed viruses were also measured in splenocytes from mice immunized with Ad5-tet-M2NP, but not Ad5-null (a negative control vector).

Immunization with Ad5-tet-M2NP induced strong CD8 and CD4 T-cell responses specific to M2 and NP epitopes. Substantial numbers of M2- and NP-responsive “effector memory” T cells were maintained in the murine spleen 8 months post-immunization, revealing a strong, long-lived T cell memory response formed by the Ad5-tet-M2NP.

## Results

### Generation of B and T-cell epitope-enriched NP and M2 consensus sequences

**Fig 1:**
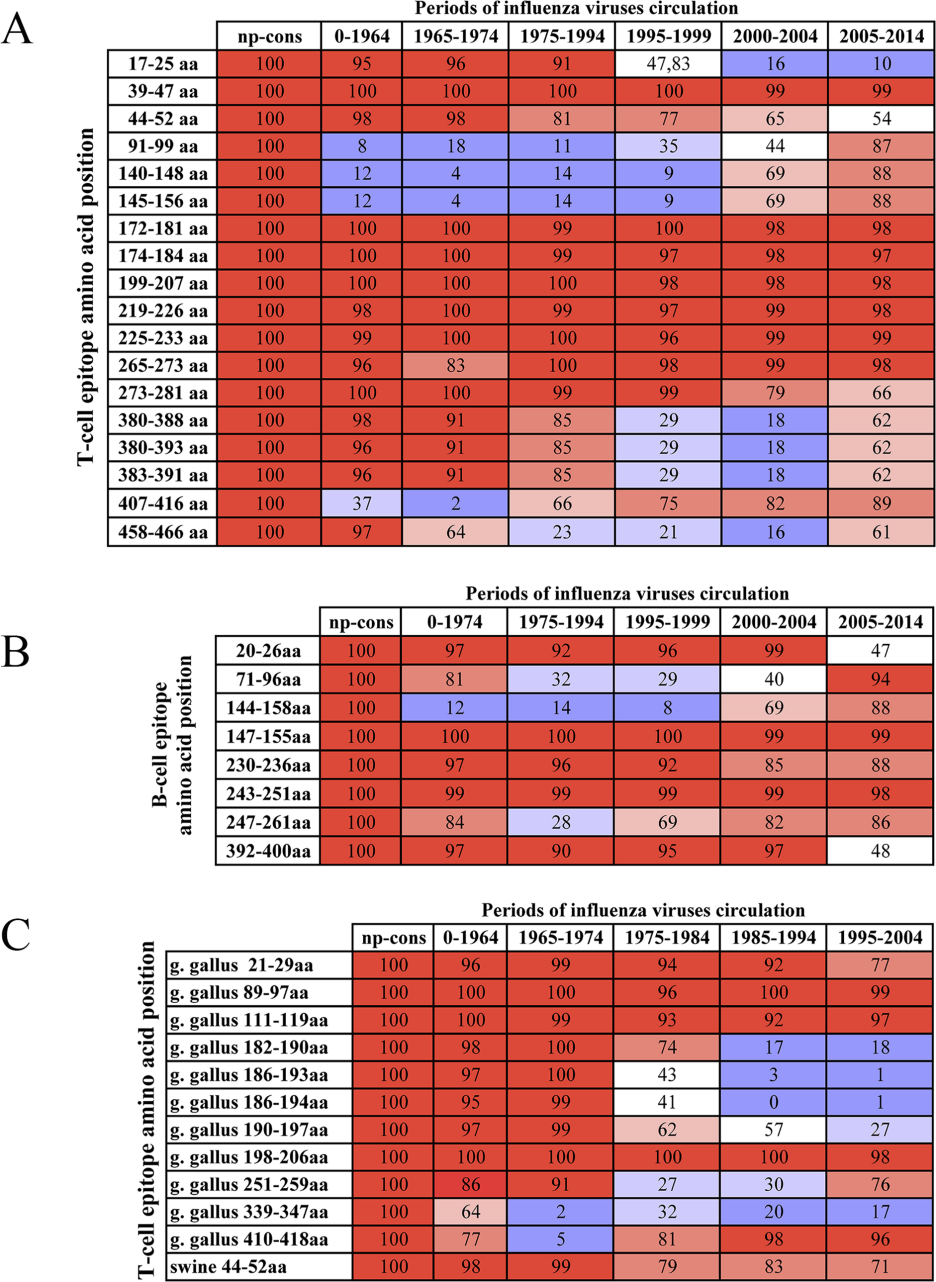
Percentage of the presence of known T and B-cell epitopes in nucleoprotein sequences of influenza viruses that circulated before 2014. The percentage of influenza viruses that have a 100% match between epitope sequences and viral sequence. A–T-cell epitopes that are specific to human viruses; B–B-cell epitopes that are specific to human viruses; C–T-cell epitopes that are specific to duck and swine viruses. Epitope sequences were aligned with NP sequences of influenza virus using the IEDB service (http://www.iedb.org/). Influenza virus sequences (all human viruses up to 2014) were obtained from the NCBI database (http://www.ncbi.nlm.nih.gov/genomes/FLU/Database/nph-select.cgi?go=database). Percentage of the presence of known T and B-cell epitopes in nucleoprotein sequences of influenza viruses in colors: red– 91–100%; pink– 71–90%; light pink– 61–70%; wight– 41–60%; light blue– 21–40%; blue– 0–20%.

**Fig 2:**
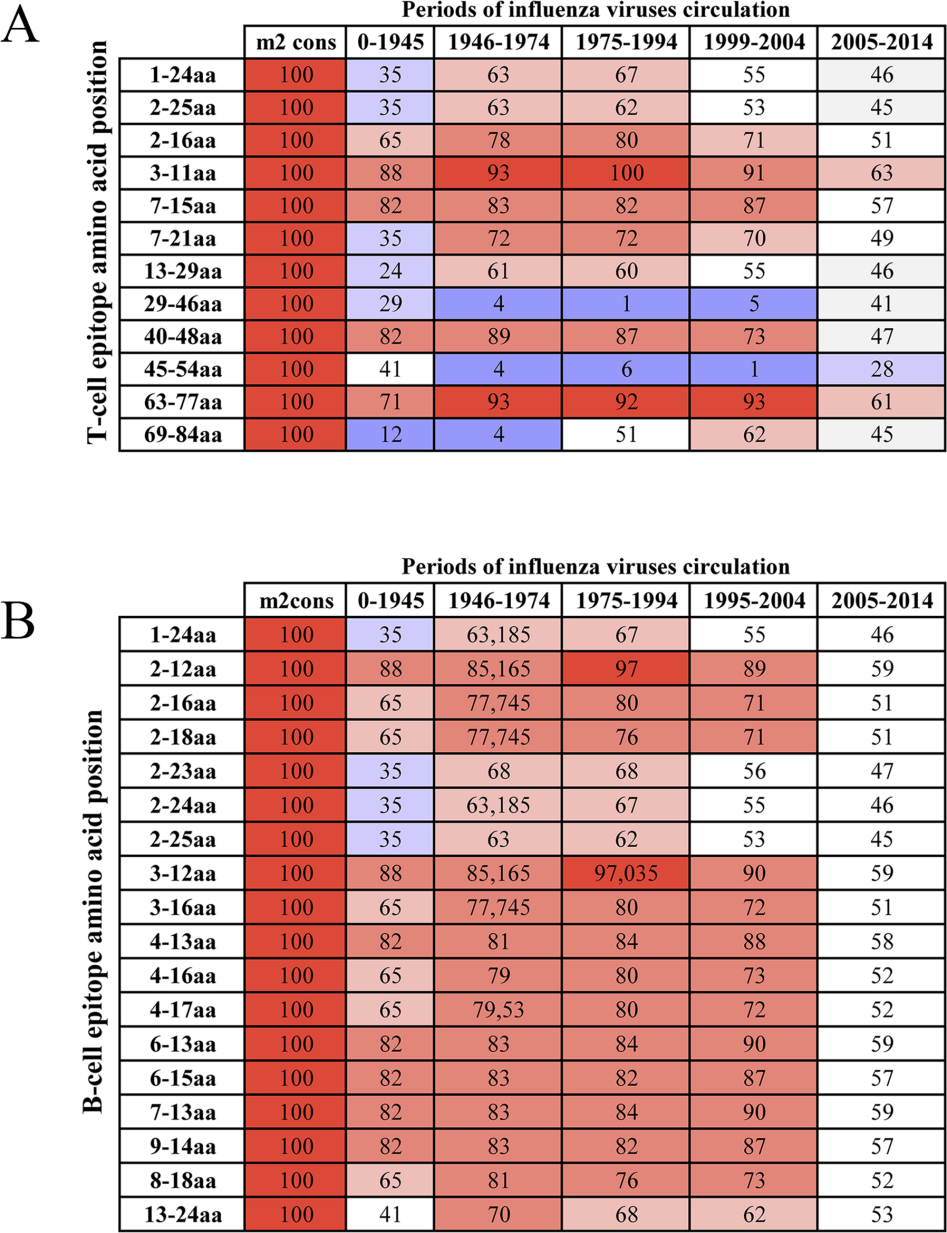
Percentage of the presence of known T and B-cell epitopes in M2 sequences of influenza viruses that circulated before 2014. The percentage of influenza viruses that have a 100% match between epitope sequences and viral sequence. A–T-cell epitopes; B–B-cell epitopes. Epitope sequences were aligned with M2 sequences of influenza virus using the IEDB service (http://www.iedb.org/). Influenza virus sequences (all human viruses through 2014) were obtained from NCBI (http://www.ncbi.nlm.nih.gov/genomes/FLU/Database/nph-select.cgi?go=database). Percentage of the presence of known T and B-cell epitopes in nucleoprotein sequences of influenza viruses in colors: red– 91–100%; pink– 71–90%; light pink– 61–70%; wight– 41–60%; light blue– 21–40%; blue– 0–20%.

It is generally believed that influenza virus M2 and NP proteins are highly conserved. We analyzed M2 and NP sequences with respect to changes in the sequences of known T and B-cell epitopes (Figs 1A–1C, 2A and 2B). For this analysis, we used all known viruses that were obtained from humans prior to 2014. The epitope sequences were obtained from IEDB (http://www.iedb.org/). We have determined that any naturally occurring protein has few known epitopes and may have no 100%-matching epitopes at all. Therefore, in theory, immunization by any natural antigen may not work due to the eventual emergence of a strain that does not have any epitopes contained in the vaccine antigen.

Next, we divided all the sequences of influenza virus NP or M2 that were obtained from humans before 2014 into sets of approximately 100 sequences, in accordance with their chronological collection. The number of sequences in each set was restricted by the completeness of the time period, e.g. a whole year, in which corresponding viruses were collected.

During the analysis of the epitopes of these grouped sequences, it was identified that in every period, viruses have one or several epitopes, but never not all of them. For example, only 93% of influenza viruses that circulated from 1999–2004 had T-cell epitope PSTEGVPESMREEYR (63–77 аа) in the M2 protein (Fig 2A); other T-cell epitopes were found in fewer viruses (Figs 1A, 1C and 2A). In our analysis, we also found that fewer than 50% of influenza viruses from 2005–2014 had known B and T-cell epitopes in M2 (Fig 2A and 2B). This fact may provide evidence that mutations have occurred in the M2 structure that caused changes in antigenic determinants and T-cell epitopes. This may likely have happened due to the emergence of new influenza strains that transferred from birds. However, we do not have sufficient data with regard to avian influenza virus epitopes to validate this suggestion.

To increase the likelihood of including known epitopes in the vaccine, we created NP and M2 consensus sequences based on viruses that were obtained from humans before 2014. While creating consensus sequences, we intentionally introduced changes in the amino acid content of proteins that could enhance the presence of epitopes. Basic principles of creating of epitopes enriched sequences we have provided in section «Development of optimal amino acid sequences of M2 and NP antigens» of «Materials and methods». The obtained sequences of both antigens consist of epitopes, indicated as 100% in columns «np-cons» и «m2-cons» of S1 Table. As a result, most of the known epitopes were 100%-homologous to corresponding areas of obtained sequences (Figs 1A–1C, 2A and 2B and S1 Table). Ultimately, the consensus sequence for M2 contained 12 of 21 T-cell epitopes and 18 of 63 B-cell epitopes (column «m2 cons» in S1 Table). The NP-consensus sequence contained 30 of 53 T-cell epitopes and 8 of 15 B-cell epitopes (column «np cons» in S1 Table).

The obtained sequence for NP antigen contained 3 fully homologous conserved epitopes that had been found by Muñoz-Medina J.E. et al [20]. The NP sequence also contained a few additional highly conserved epitopes: specific for humans, conserved among influenza viruses H1N1, H3N2, H5N1, was epitope ILRGSVAHK; conserved among influenza viruses H1N1, H3N2, H5N1, specific for birds, was epitope KTGGPIYRR, as well as additional less-conserved epitopes. [21]. The obtained sequence of M2-antigen also contained highly conserved antibody epitopes: MSLLTEVETPIRNEWGCRCNDSSD (1–24 аа) and SLLTEVETPIRNEWGCRCNDSSD (2–25 аа) [21, 22].

**Fig 3:**
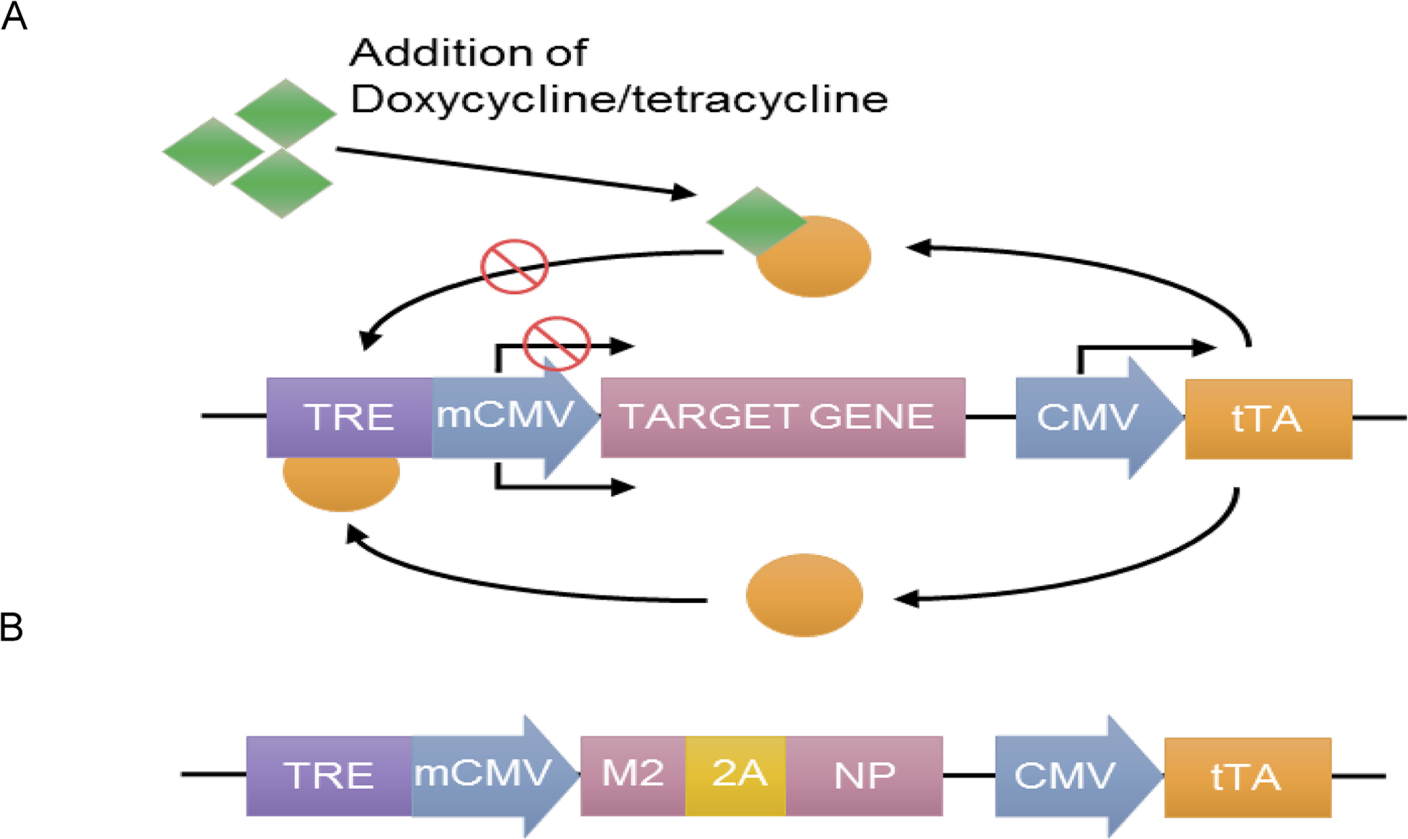
**Schematic diagram of expression system Tet-off (A) and developed genetic construction (B).** TRE–tetracycline response element, mCMV–minimal CMV promoter, M2 –M2 protein gene, 2A –nucleotide sequence that codes foot-and-mouth disease virus 2A peptide, NP–NP protein gene, CMV–human cytomegalovirus promoter, tTA–tetracycline-controlled transactivator. Tet-off system consists of two expression cassettes. The first of them contain gene of interest under the control of mCMV. Additionally, this cassette contains TRE. For the activation of expression of gene of interest tTA should bind to the TRE. The second expression cassette contain tTA gene under the control of CMV. Tetracycline and doxycycline bind to tTA and block expression of gene of interest.

To study the effect of antigens with the obtained consensus sequences of M2 and NP, these were included in a recombinant vector based on human adenovirus serotype 5 (rAd). For this purpose, the amino acid sequence of the NP protein was modified by the removal of a nuclear localization signal. The literature indicates that such a removal prevents NP protein transport to the cell nucleus and enhances its immunogenicity through effective processing, forming T-cell epitopes in the cytoplasm of antigen-presenting cells. The obtained T-cell epitopes bind with TAP proteins (transporter associated with antigen processing), and they are then transported to the endoplasmic reticulum where they join with molecules of MHC class I and are secreted by the Golgi apparatus to the surface of the cell for presentation to T-lymphocytes [23, 24]. Consensus sequences of the M2 and NP proteins were fused to each other through sequence that encode self-cleaving peptide 2A. We also additionally inserted the Tet-regulating element (Fig 3A and 3B), which enabled us to successfully propagate the Ad5-tet-M2NP vector at a large volume, thereby avoiding toxic effects of the full-length M2 in HEK 293 cells [25, 26, 27].

### In vitro characterization of rAd-expressing influenza M2 and NP

**Fig 4:**
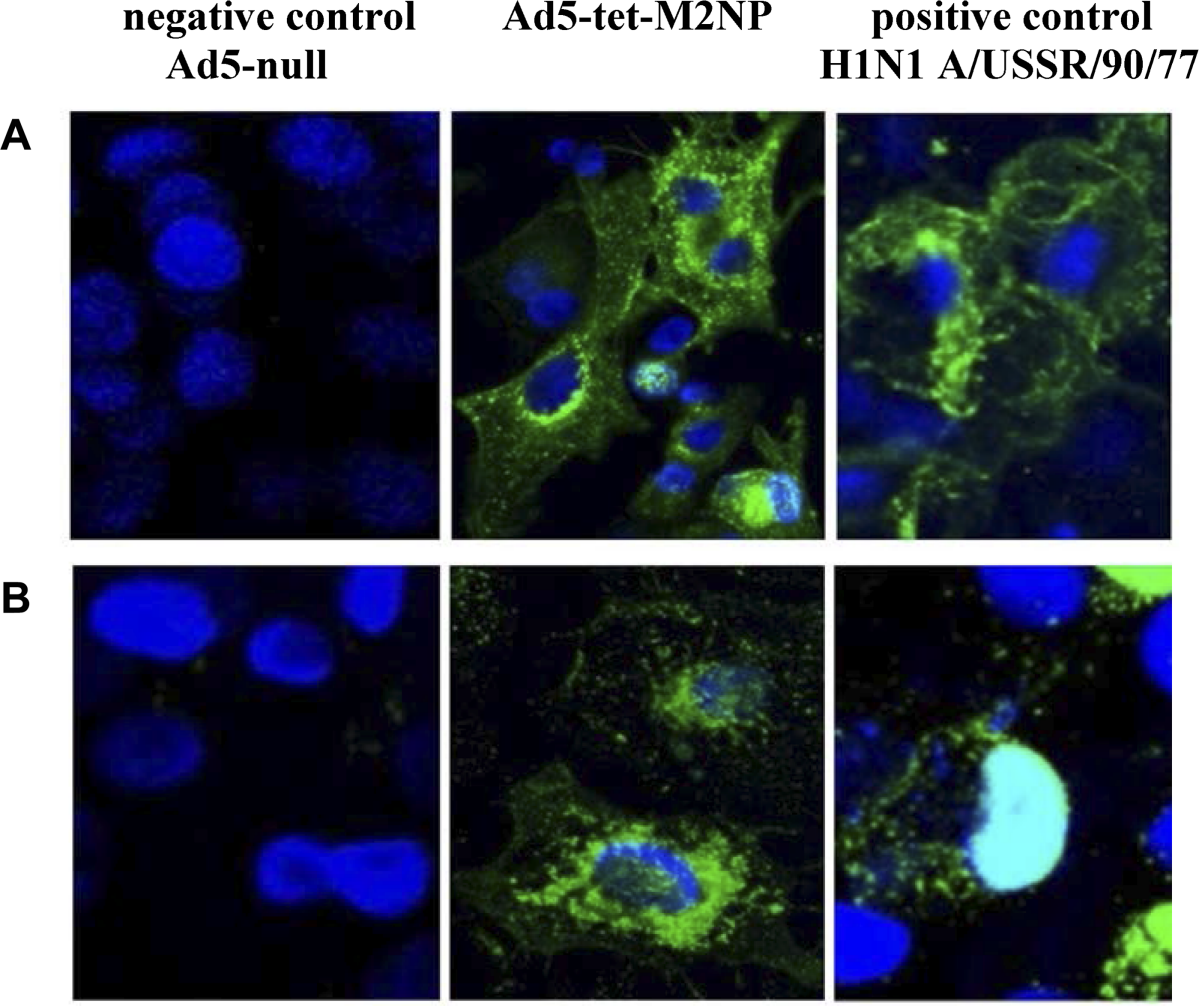
Expression of M2 and NP proteins in A549 cell line transduced with Ad5-tet-M2NP. (**A**). Cells were labeled for the M2-protein using M2-specific monoclonal antibodies and Dylight 488 (**B**). Cells were labeled for the NP-protein using NP-specific monoclonal antibodies and Dylight 488. Vertical lines: left—negative control–cells transduced with Ad5-null (“empty” vehicle); middle—cells transduced with Ad5-tet-M2NP; right—positive control–cells were infected with 869 influenza virus A/USSR/90/77 (H1N1).

The expression of the M2 and NP genes as part of the recombinant adenovirus Ad5-tet-M2NP was evaluated by immunofluorescence microscopy with the use of murine monoclonal antibodies to the M2 (14C2) and NP (B248M) proteins, and secondary antibodies that were marked with the fluorescent dye Dylight 488. A549 cells were transduced with Ad5-tet-M2NP. As a negative control, we used cells that had been transduced with recombinant adenovirus without any transgene—Ad5-null. As a positive control, we used cells that had been infected with influenza virus A/USSR/90/77 (H1N1). 48 hours after transduction, the cells were fixed with acetone and were incubated with antibodies. The evaluation of results of immunohistochemistry was performed by confocal microscopy (Fig 4A and 4B). Through antibodies that were marked with a fluorescent dye, the M2 (Fig 4A) and NP (Fig 4B) proteins were visualized in green. Cell nuclei were visualized in blue via the DAPI (4',6-diamidino-2-phenylindole) dye.

The expression of the M2 and NP genes within the Ad5-tet-M2NP vector was confirmed (Fig 4A and 4B). Moreover, cytoplasmic localization of the NP protein was observed in cells that had been transduced with Ad5-tet-M2NP (Fig 4B), as distinguished from the positive control where the NP protein was located in the cell nucleus (Fig 4B); this in its turn attests to the successful removal of the nuclear localization signal of the NP protein, which makes it accessible for effective processing in the cytoplasm.

### M2- and NP-specific serum antibody response and T cell responses

To evaluate the immune response of the Ad5-tet-M2NP in vivo and to determine the immunization dose of Ad5-tet-M2NP, we examined M2- and NP-specific serum antibody response and T cell responses at thirty days post-immunization (challenge time point). For this we administered Ad5-tet-M2NP intranasally to Balb/c mice. Two doses of Ad5-tet-M2NP were tested: 10^8^ PFU/mouse and 10^7^ PFU/mouse (two groups, 10 mice per group). Control groups (two groups, 10 mice per group) were given the same volume of recombinant adenovirus Ad5-null at a dose of 10^8^ PFU/mouse or PBS. Thirty days post-immunization, serum and spleens were collected from all animals and examined for M2- and NP-specific serum antibody response and T cell responses, respectively.

**Fig 5:**
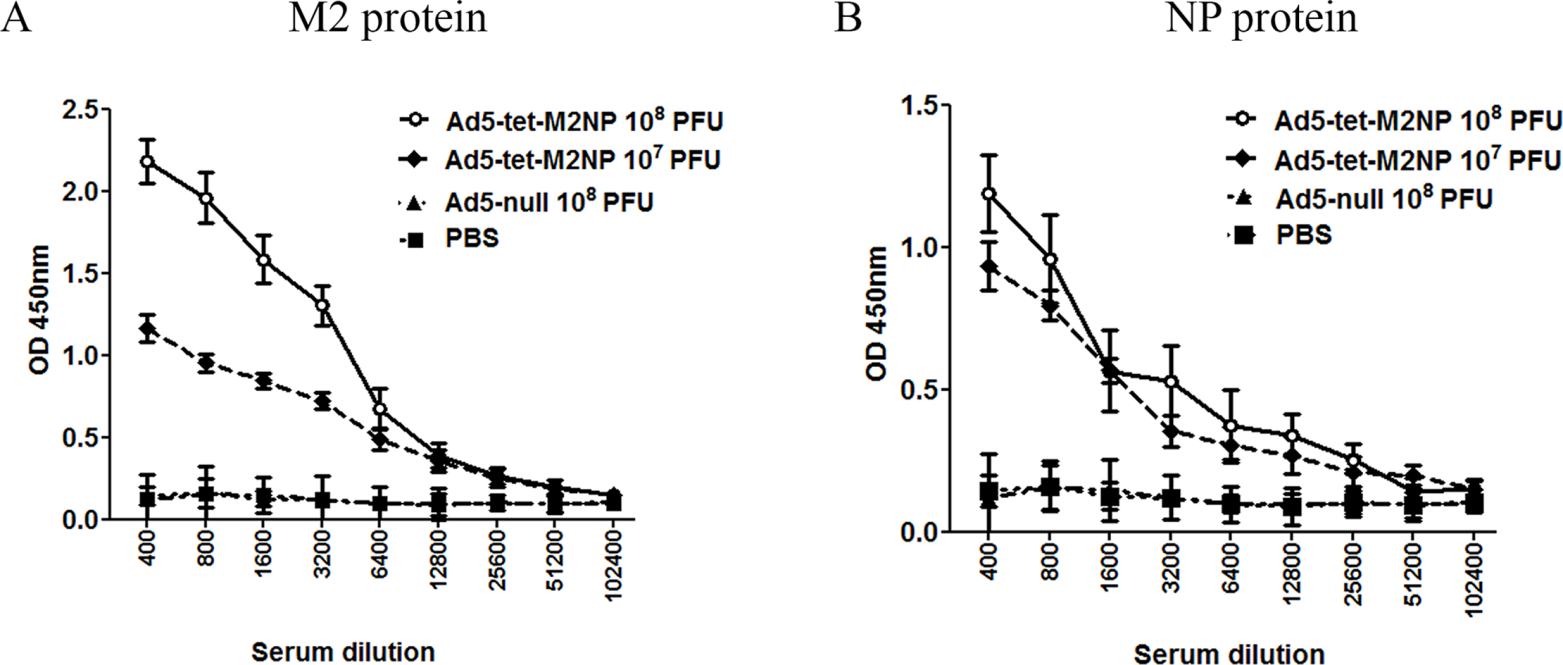
ELISA titration for M2- and NP-binding IgG antibodies in the sera of mice at the 30^th^ day after immunization with Ad5-tet-M2NP or Ad-null vectors. Mice were immunized intranasally using 10^8^ PFU or 10^7^ PFU doses of Ad5-tet-M2NP, 10^8^ PFU Ad-null, or PBS. Mouse sera were collected 30 days post-immunization. Titration of the sera was performed in ELISA plates pre-coated with synthetic M2 (**A**) or recombinant NP (**B**) proteins. *X*-axis–serum dilution; *Y*-axis–optical density (*λ* = 450 nm). Data are represented as the mean **± SD** for 10 mice in the experimental group.

Serum was collected and examined for M2- and NP-binding IgG-antibodies using ELISA with synthetic M2 or recombinant NP proteins. According to the obtained data (Fig 5A and 5B), intranasal administration of Ad5-tet-M2NP induced strong antibody responses to both the M2 and NP antigens. The titers of serum IgG-antibodies specific for M2 and NP proteins were estimated as 25,600 and 12,800, respectively. The M2-specific antibody response to the 10^7^ PFU dose of Ad5-tet-M2NP was substantially lower than to 10^8^ PFU, while the NP-specific antibody-responses to both doses were practically identical. No M2- or NP-specific antibodies were detected in the sera of control mice that received either intranasal Ad5-null vector or PBS.

**Fig 6:**
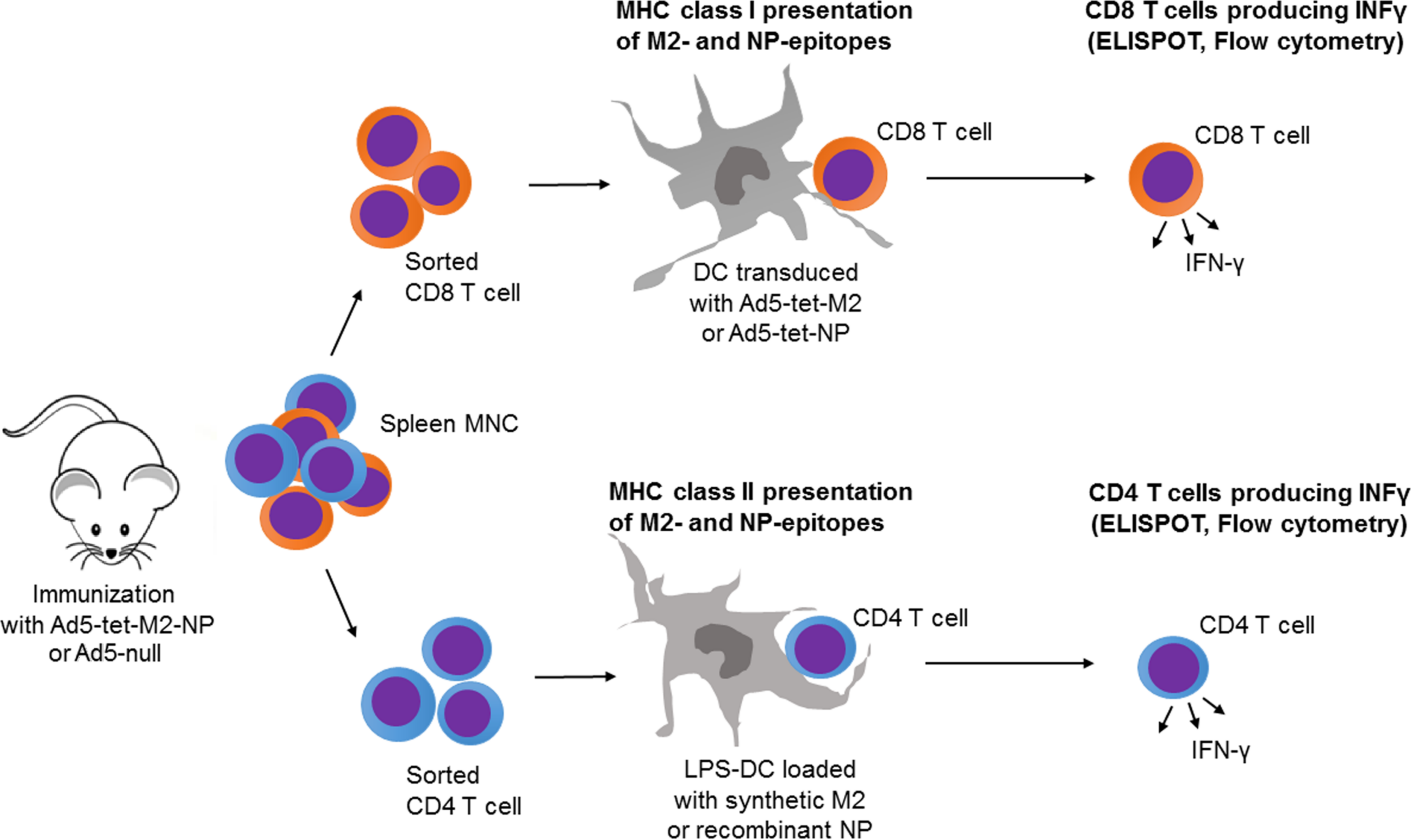
Schematic diagram for quantification of purified CD8 and CD4 T cell responses to M2 and NP epitopes presented on the surface of DCs or LPS-DCs in the context of MHC class I or MHC class II. Mice were pre-immunized with Ad5-tet-M2NP or Ad-null, 1 month or 8 months after this their spleens were aseptically collected, and the mononuclear cell fraction was obtained using ficoll-plaque gradient centrifugation. 99%-pure CD8 and CD4 cell suspensions were obtained using FACS-sorting. Syngenic bone marrow-derived dendritic cells (DCs) were differentiated in 7-day cultures with GM-CSF (granulocyte macrophage colony-stimulating factor). For presentation of antigens in the context of MHC class I, dendritic cells were transduced with recombinant Ad5-tet-M2, Ad5-tet-NP, or Ad5-null (100 PFU per cell).

The numbers of M2- and NP-reactive T cells were counted in the spleen via enzyme-linked immunospot (ELISPOT) and flow cytometry as it is described in «Materials and Methods». Purified CD4 or CD8 T cells, or splenic mononuclear cells from immunized mice were reactivated with DCs preloaded with the synthetic M2 (MSLLTEVETPIRNEWGCRCNDSSD) or recombinant NP proteins for the MHC-II-restricted, or transduced with either Ad5-tet-M2, Ad5-tet-NP, Ad5-null, or none for MHC-I-restricted antigen presentation (Fig 6).

**Fig 7:**
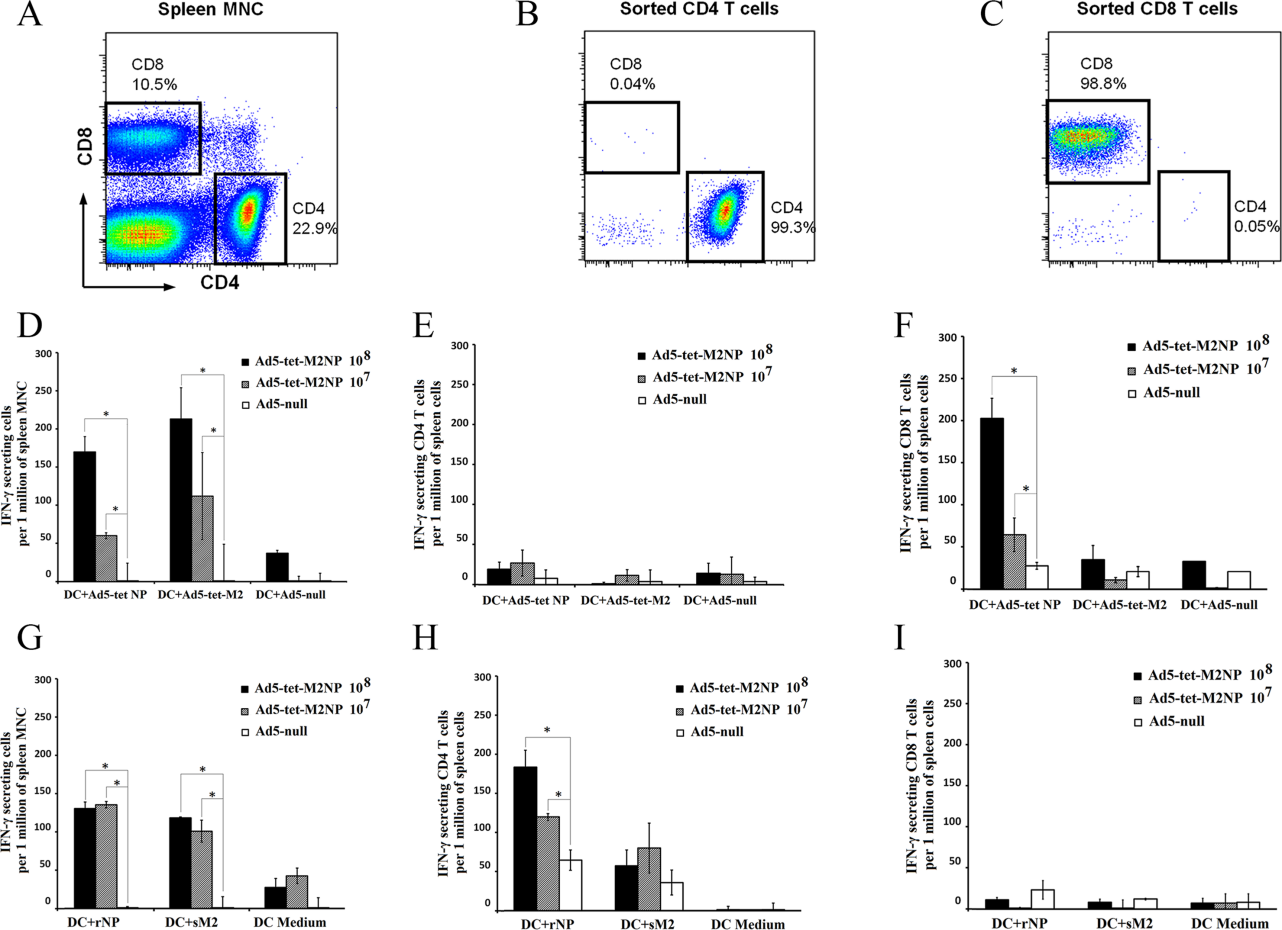
Quantification of interferon-γ secreting M2- or NP-specific T cells in the spleens of mice immunized with Ad5-tet-M2NP. BALB/c mice received a single intranasal immunization with either 10^7^ PFU or 10^8^ PFU doses of Ad5-tet-M2NP or 10^8^ PFU Ad5-null (“empty vector”). Thirty days post-immunization, mouse spleens were aseptically collected and the MNC fraction was obtained using ficoll-plaque gradient centrifugation. 99%-pure CD8 and CD4 cell suspensions were obtained using cell sorting by a BD FACSAria II (A-C). Syngenic dendritic cells (DCs) were differentiated in 7-day bone marrow cultures with GM-CSF. For the presentation of antigens in the context of MHC class I (D, E, F), dendritic cells were transduced with recombinant Ad5-tet-M2 or Ad5-tet-NP, or Ad5-null (100 PFU per cell). For the presentation of antigens in the context of MHC class II (G, H, I), DCs were pre-activated with LPS (1 μg/ml) (LPS-DC) and then loaded with either 50 μg/ml synthetic M2 (sM2) or 5 μg/ml recombinant NP (rNP) antigens. Splenic MNCs (D, G) or purified CD8 (F, I) or CD4 (E, H) cells were co-cultured in duplicate over 18 hrs with antigen-presenting DCs or LPS-DCs before the numbers of interferon-γ-secreting T cells were counted using ELISPOT. Numbers of spots are shown as the mean ± SD (per 1 million splenic MNCs), after deduction of the baseline spots found in the negative control group (mice that received a sham immunization, i.e., intranasal PBS). Asterisks are shown for significant differences (p<0.05) in comparison to the control group of mice immunized with Ad5-null (Mann–Whitney–Wilcoxon nonparametric test).

Data presented in Fig 7 (Fig 7A, 7D and 7G) indicate that intranasal immunization of mice with Ad5-tet-M2NP induced strong systemic T cell responses specific to M2- and NP-antigens. Spleens harvested from the immunized mice 30 days post-immunization contained 100–200 M2- or NP-reactive T cells per 1 million splenic MNCs, which was equivalent to 10,000–20,000 M2- or NP-reactive T cells per spleen. In splenic MNCs from mice immunized with Ad5-tet-M2NP, 100–150 cells (per 1 million MNCs) responded by interferon-gamma (IFN-γ)-secretion to M2-epitopes presented on DCs in the context of MHC class I (DC transduced with Ad5-tet-M2 (Fig 6)). Such T cell responses to intranasal administration of 10^8^ PFU were twice as high as those to the 10^7^ PFU dose of Ad5-tet-M2NP (Fig 7D). Similarly, 60–160 IFN-γ-secreting cells (per 1 million splenic MNCs) were detected when MNCs from the Ad5-tet-M2NP-immunized mice were re-activated in vitro with the DCs expressing NP-epitopes in the context of MHC class I (DC transduced with Ad5-tet-NP (Fig 6)). The number of NP-reactive IFN-γ-secreting cells in the spleen was ~3 times higher when mice were immunized with the 10^8^ PFU dose compared to the 10^7^ PFU dose of Ad5-tet-M2NP. It should be noted that neither Ad5-tet-M2NP immunization doses nor Ad5-null immunization induced Ad5-specific IFN-γ-secreting cells, which must be revealed with the use of Ad5-null-transduced DCs. Regardless of the immunogenic dose used, mice that received 10^7^ or 10^8^ PFU Ad5-tet-M2NP accumulated approximately 100–110 M2- and 120–130 NP-reactive IFN-γ-secreting cells (Fig 7G), recognizing these antigens in the context of MHC class II (DC-LPS (DC-lipopolysaccharide) loaded with M2 or NP (Fig 6). When studying splenic mononuclear cells (MNCs) in ELISPOT assays, it is impossible to establish the nature of interferon-γ-secreting cells (IFN-γ-secreting cells), which can be T-, NKT (natural killer T cell)- or NK (natural killer) cells.

We therefore studied highly purified CD4 T cells and CD8 T cells using FACS-sorted cell populations, each with 99% purity (Fig 7A, 7B and 7C), to specify the nature of the IFN-γ-secreting cells induced by Ad5-tet-M2NP immunization. Purified CD4 T cells responded to in vitro re-stimulation by DCs loaded with synthetic M2 or recombinant NP antigens (Fig 7H), but did not respond to DCs transduced with Ad5-tet-M2 or Ad5-tet-NP (Fig 7E). Conversely, purified CD8 T cells from spleens of Ad5-tet-M2NP immunized mice responded with IFN-γ secretion to NP-, but not M2-antigenic epitopes presented on DCs in the context of MHC class I (Fig 7F), and no CD8 T cell response was observed to NP- and M2 presented on DC-LPS in the context of MHC class II (Fig 7I).

It must be noted that, in contrast to NP, the responses of purified CD4 T cells and CD8 T cells to M2-derived epitopes were low or even negligible. This low responsiveness was observed with purified T cells only, whereas splenic mononuclear cell suspensions showed strong responses to M2 in the context of both MHC classes I and II (see respective bars on Fig 7D and 7G).

Based on the data obtained, the higher of the two doses of Ad5-tet-M2NP was selected for further tests and challenge experiments (i.e., 10^8^ PFU/mouse).

### Ad5-tet-M2NP induce long lasting M2- and NP-specific immune responses

Eight months study was dedicated to examining of the long-term M2- and NP-specific immunological memory which is represented by T cells. At the same time point the serum was collected, where antibodies were detected. For this we intranasally administered 10^8^ PFU/mouse of Ad5-tet-M2NP to 10 Balb/c mice. The second group of mice (10 animals) were intranasally administered with the same volume of recombinant adenovirus Ad5-null at a dose of 10^8^ PFU/mouse. Eight months after immunization, serum and spleens were collected from all animals and examined for M2- and NP-specific serum antibody response and T cell responses, respectively.

**Fig 8:**
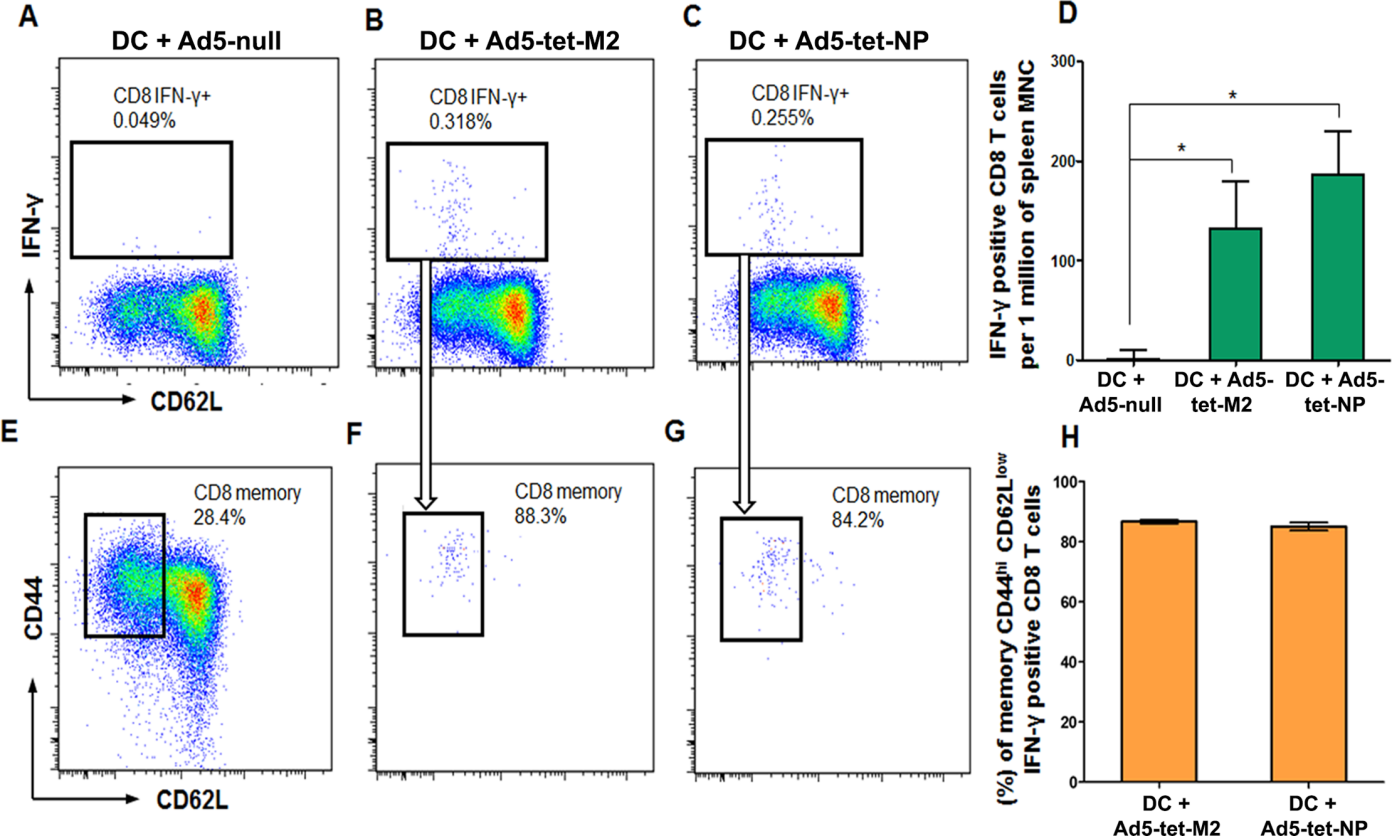
Flow cytometry analysis of interferon-γ-secreting M2- and NP-recognizing CD8 T cells and “effector memory” CD44^high^CD62L^low^ CD8 T cells in the spleens of BALB/c mice, 8 months post-immunization with Ad5-tet-M2NP. Splenic mononuclear cells obtained from BALB/c mice 8 months after a single intranasal immunization with 10^8^ PFU of Ad5-tet-M2NP were co-cultured for 18 hrs with syngenic dendritic cells transduced with either Ad5-null (“empty” vehicle, **A**), Ad5-tet-M2 (**B**) or Ad5-tet-NP (**C**), and then analyzed for intracellular IFN-***γ*** using a BD FACSAria II flow cytometer. **A**-**C** show dot plots gated for CD8 T cells producing IFN-***γ*** in respective cultures. **D**–numbers of IFN-***γ***-producing CD8 T cells per 1 million splenic MNCs are shown. Gated areas in dot plots show: (**E**) a portion of “effector memory” CD44^high^CD62L^low^ CD8 T cells among all splenic CD8 cells; (**F**) “effector memory” CD44^high^CD62L^low^ CD8 T cells specifically responding to M2-epitopes or (**G**) to NP-epitopes. **H**–percent of IFN-***γ***-producing “effector memory” CD44^high^CD62L^low^ CD8 T cells specifically responding to M2-epitopes or NP-epitopes in the population of all CD8 T cells responding to M2-epitopes or NP-epitopes.

MNCs obtained by FACS-sorting were studied using flow cytometry. MNCs from spleens of Ad5-tet-M2NP-immunized mice were co-incubated with the Ad5-tet-M2-transduced DCs or the Ad5-tet-NP-transduced DCs. As a result of in vitro re-activation of T cells with M2- or NP-epitopes presented on DCs in the context of MHC class I, there were approximately 150 and 190 IFN-γ-producing CD8 T cells (per 1 million splenic MNCs) recorded that recognized M2- and NP-epitopes, respectively (Fig 8B–8D). In co-cultures of MNCs from the spleens of Ad5-tet-M2NP-immunized mice with the control Ad5-null-transduced DCs, there were negligible numbers of IFN-γ-producing CD8 T cells recorded (Fig 8A and 8D).

Data obtained showed that a single intranasal immunization with 10^8^ PFU of Ad5-tet-M2NP conferred long-term M2- and NP-specific T cell memory. Eight months post immunization, in the spleens of immunized mice there were more than 100 CD8 T cells (per 1 million splenic MNCs) specifically responding to either M2- or NP-antigens by the production of IFN-γ (Fig 8D), and more than 80% of these cells were represented by effector memory CD44posCD62neg CD8 T cells (Fig 8E–8H).

**Fig 9:**
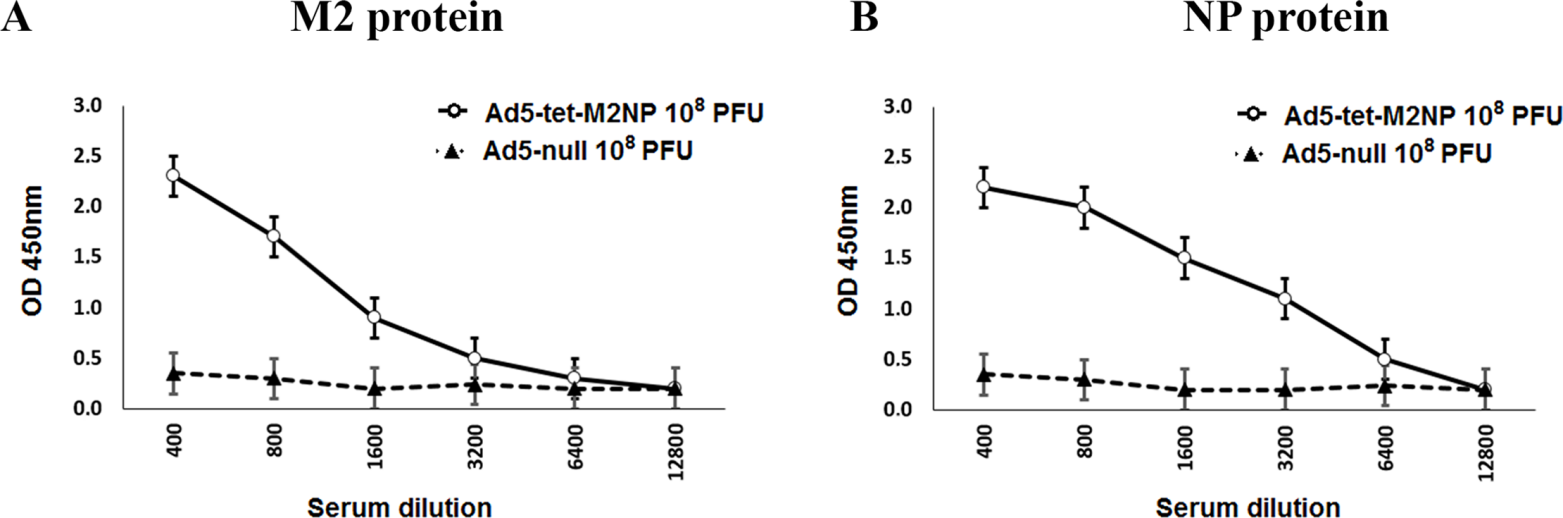
ELISA titration for M2- and NP-binding IgG antibodies in the sera of mice, 8 months post-immunization with Ad5-tet-M2NP. Mice were immunized intranasally using 10^8^ PFU of Ad5-tet-M2NP and 10^8^ PFU of Ad-null. Mouse sera were collected 8 months post-immunization. Titration of the sera was performed in ELISA plates pre-coated with synthetic M2 (**A**) or recombinant NP (**B**) proteins. *X*-axis–serum dilution; *Y*-axis–optical density (*λ* = 450 nm). Data are represented as the mean **± SD** for 10 mice in the experimental group.

Serum was examined for M2- and NP-binding IgG-antibodies using ELISA that was described above for 1-month studies. According to the obtained data (Fig 9A and 9B), Ad5-tet-M2NP induced strong antibody responses to both the M2 and NP antigens. The titers of serum IgG-antibodies specific for M2 and NP proteins were estimated as 6,400 and 3,200, respectively. No M2- or NP-specific antibodies were detected in the sera of control mice that received Ad5-null vector (Fig 9A and 9B).

### Immunization with Ad5-tet-M2NP induces antibody and T cell responses that are cross-reactive to different subtypes of influenza viruses belonging to 3 different clades from 3 different hosts

M2 and NP sequences encoded by the Ad5-tet-M2NP vector were designed as consensus M2- and NP-sequences from different influenza virus subtypes, which were then corrected by analysis and inclusion of dominant worldwide sequences from the last 100 years for B and T cell epitopes, experimentally confirmed for different influenza virus subtypes from 3 different hosts. It was therefore important to determine the cross-reactivity of antibody and T cell responses induced by Ad5-tet-M2NP for different influenza virus subtypes.

**Fig 10:**
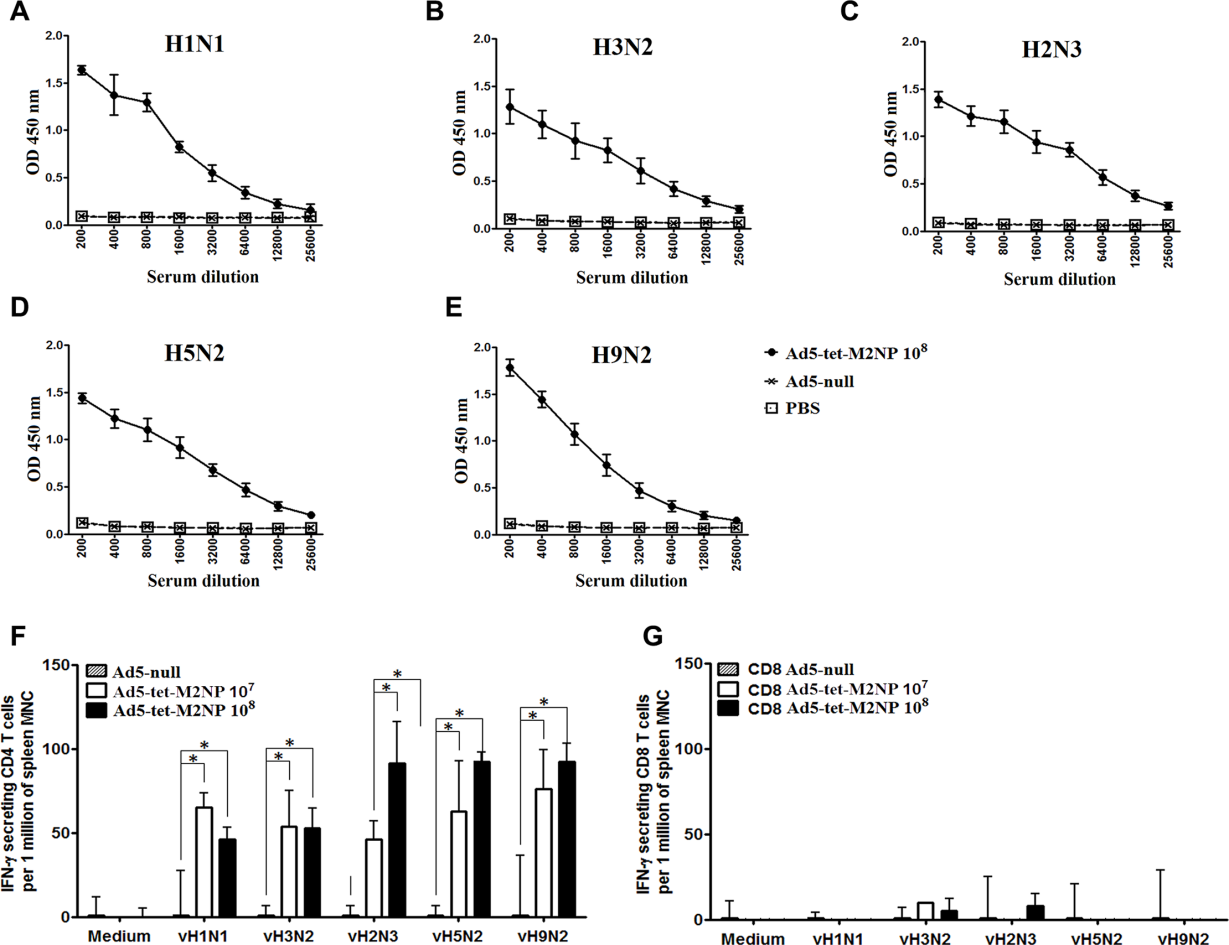
Broad-spectrum cross-reactivity of serum IgG antibodies and splenic T cells of mice immunized with Ad5-tet-M2NP towards lysates of 5 different heterosubtypes of influenza viruses. Mice were immunized intranasally using 10^8^ PFU doses of Ad5-tet-M2NP or Ad-null. **A**-**E** show sera titration curves by ELISA. Mouse sera were collected 30 days post-immunization. Titration of the sera was performed in ELISA plates pre-coated with lysates of either H1N1 (A/USSR/90/77), H2N3 (A/BlackDuck/NewJersey/1580/78), H3N2 (A/Aichi/2/68), H5N2 (A/Duck(Mallard)/Pennsylvania/10218/84) or H9N2 (A/Swine/Hong Kong/9A-1/98) influenza viruses. *X*-axis–serum dilution; *Y*-axis–optical density (*λ* = 450 nm). Data are represented as the mean **± SD** for 10 mice in each experimental group. **F** and **G** show purified CD4 and CD8 T cell responses to lysates of the 5 heterosubtypes of influenza viruses listed above. Thirty days post-immunization with intranasal 10^7^ PFU or 10^8^ PFU doses of Ad5-tet-M2NP, or 10^8^ dose of Ad-null, mice were sacrificed, spleens were aseptically collected and purified CD4 and CD8 T cells were sorted using a BD FACSAria II. Purified CD4 and CD8 T cells were co-cultured in duplicate for 18 hours with LPS-DCs loaded with one of the viral lysates, as indicated. Counts of IFN-γ-secreting cells were defined using ELISPOT. Numbers of spots are shown as the mean ± SD (per 1 million splenic MNCs), after deduction of spots found in the control group (mice that received “sham” immunization with Ad5-null). Asterisks are shown for significant differences (p<0.05) in comparison to the control group of mice immunized with Ad5-null (Mann–Whitney–Wilcoxon nonparametric test).

Immune sera harvested from mice immunized with either Ad5-tet-M2NP (10^8^ PFU, intranasally) or Ad5-null (10^8^ PFU, intranasally) were examined by ELISA wherein lysates of H1N1 (A/USSR/90/77), H9N2 (A/Swine/Hong Kong/9A-1/98), H3N2 (A/Aichi/2/68), H2N3 (A/BlackDuck/NewJersey/1580/78), or H5N2 (A/Duck(Mallard)/Pennsylvania/10218/84) viruses were used as antigens. Data obtained indicated that tested sera from mice immunized with Ad5-tet-M2NP contained high levels of antibodies cross-reactive to all 5 viruses used (Fig 10A–10E). Titers of serum IgG-antibodies capable of binding viral lysate proteins varied between 12,800 and 25,600. Sera from control mice, immunized with Ad-null, did not react with the viral lysates used.

The cross-reactivity of T cells was examined in ELISPOT assays with the use of LPS-DCs pre-loaded with lysates of H1N1 (A/USSR/90/77), H9N2 (A/Swine/Hong Kong/9A-1/98), H3N2 (A/Aichi/2/68), H2N3 (A/BlackDuck/NewJersey/1580/78), or H5N2 (A/Duck(Mallard)/Pennsylvania/10218/84) influenza viruses. As shown in Fig 10F, purified CD4 T cells reacted strongly to viral antigens from all 5 virus subtypes preloaded in LPS-DCs. Purified CD8 T cells showed no reaction to LPS-DCs expressing different influenza viral proteins in the context of MHC class II (Fig 10G). The observed absence of CD8 T cell responses was reasonable and expected, as it showed the absence of any cross-presentation of the loaded viral proteins to MHC class I molecules of LPS-DCs.

Thus, according to the data obtained (Fig 10A–10G), a single intranasal immunization of BALB/c mice with Ad5-tet-M2NP induced intense production of M2- and NP-specific serum antibodies and T cells responses, and both types of responses demonstrated an outstanding wide-ranging cross-reactivity to viral proteins from influenza viruses belonging to 5 different virus heterosubtypes from 3 different hosts.

### Protection of mice from a lethal challenge with different subtypes of influenza viruses after a single intranasal immunization with Ad5-tet-M2NP

BALB/c mice were immunized via an intranasal administration of Ad5-tet-M2NP or Ad5-null, each at a dose of 10^8^ PFU per mouse. There were 10 total groups, each with 10 mice– 5 groups immunized with Ad5-tet-M2NP and 5 groups immunized with Ad5-null. Five separate challenge experiments were performed, each using one of the following influenza A virus subtypes: H9N2 (A/Swine/Hong Kong/9A-1/98), H1N1 (A/USSR/90/77), H5N2 (A/Duck(Mallard)/Pennsylvania/10218/84), H2N3 (A/BlackDuck/NewJersey/1580/78) or H3N2 (A/Aichi/2/68). All the subtypes of the viruses were adapted for mice [28].

**Fig 11:**
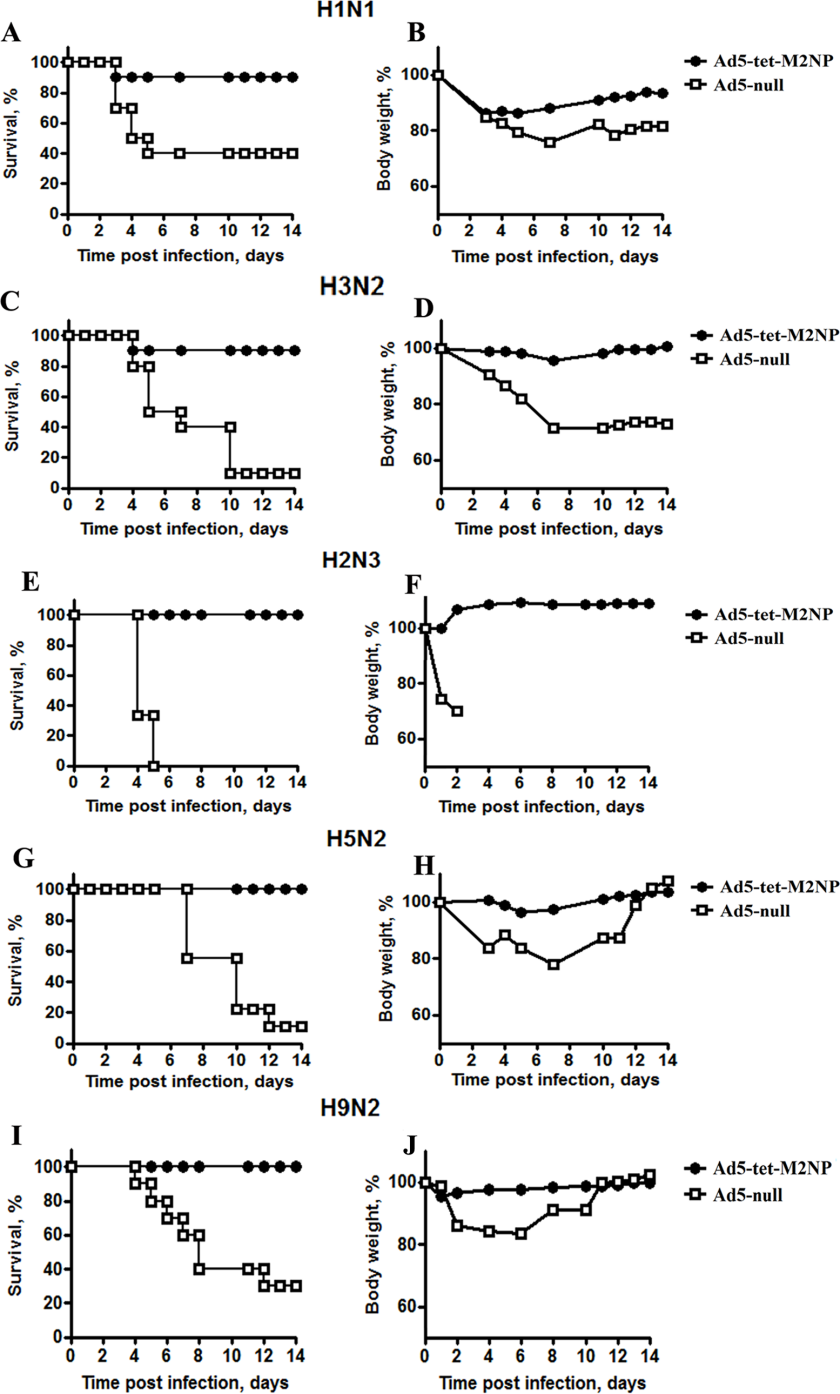
Broad protection of BALB/c mice from lethal challenge with 5 different heterosubtypes of influenza viruses by a single intranasal immunization with Ad5-tet-M2NP. Mice were immunized intranasally using 10^8^ PFU doses of Ad5-tet-M2NP or Ad-null. One month post-immunization, mice were challenged with a 10x LD_50_ dose of one of the following influenza viruses: H1N1 (A/USSR/90/77) (A, B), H3N2 (A/Aichi/2/68) (C, D), H2N3 (A/BlackDuck/NewJersey/1580/78) (E, F), H5N2 (A/Duck(Mallard)/Pennsylvania/10218/84) (G, H) or H9N2 (A/Swine/Hong Kong/9A-1/98) (I, J). Five challenge experiments were performed, each dedicated to challenge with one of the listed viruses. In each of the 5 challenge experiments, there was one group of mice (n = 10) immunized with Ad5-tet-M2NP and another group of control mice (n = 10) immunized with Ad5-null. The mouse body weight loss (morbidity) and mortality were monitored daily for 14 days post-challenge. Charts show daily dynamics of survival rate (percent) and body weight (percent of initial weight). Differences p<0.05 in comparison to the control group of mice.

Thirty days post-immunization, mice were challenged with 10x LD50 of each type of influenza virus. In each of the challenge experiments, there was one group of mice immunized with Ad5-tet-M2NP and one control group immunized with Ad5-null. Morbidity (body weight loss) and mortality were monitored twice daily for 14 days. Data are represented in Fig 11A–11J and Table 1. In all control animals immunized with the “empty” vector (Ad5-null), mice suffered from the infecting disease, which was obvious due to a substantial loss of body weight. In the control groups, the mortality rate due to challenge with different subtypes of influenza A viruses ranged between 60% and 100%.

**Table 1:** Survival rate in 5 challenge experiments with mice pre-immunized with Ad5-tet-M2NP or Ad5-null.

Groups of mice	Subtypes of influenza A viruses used for the challenge in a dose of 10 LD_50_				
	H1N1	H2N3	H3N2	H5N2	H9N2
**Ad5-tet-M2NP**	**80**	**100**	**90**	**100**	**100**
**Ad5-null**	**40**	**0**	**10**	**10**	**30**

In the groups of mice immunized with Ad5-tet-M2NP, there was neither significant morbidity nor mortality recorded during the 2 weeks post-challenge. Excitingly, after a single intranasal immunization with Ad5-tet-M2NP, mortality declined to 20% and 10% in groups challenged with 10x LD50 of H1N1 and H3N2, respectively, and to zero in all other groups, i.e., the H9N2, H5N2 or H2N3 subtypes of influenza viruses (Fig 11A, 11C, 11E, 11G and 11I and Table 1).

In all, the challenge experiments showed an extraordinarily wide-ranging efficacy of protection by the Ad5-tet-M2NP vaccine, covering 5 different heterosubtypes of influenza A virus.

## Discussion

The key challenges for creating vaccines against influenza A virus are antigenic drift and antigenic shift. Preventive effects of current influenza virus vaccines are quite specific to particular strains of influenza virus and are based generally on antibody responses to surface antigens of the virus (hemagglutinin and neuraminidase), the structures of which are the most mutable [29]. Thus, influenza virus vaccines are not able to provide protection from a wide spectrum of diverse viral strains. Therefore, to avoid outbreaks of epidemics and pandemics among human populations, modern medicine requires the development of new universal vaccines that are able to provide protection from a wide range of influenza A virus strains.

Traditionally, when designing universal vaccines against influenza A, heterosubtypic vaccines are preferred, considering nomenclature, that are based on subtypes of hemagglutinin and neuraminidase. For this, only strains of viruses that infect people are typically considered. However, it is known that new human epidemic strains of influenza viruses frequently have their origins in viruses of birds and other animals [30]. Therefore, when creating a universal vaccine, it is essential to consider that immunity must additionally be developed against viruses from other hosts.

It has been shown that highly-conserved proteins, such as M2 and NP, differ in regard not to the viral subtype, but to the ability to infect specific animal species. Hence, vaccines based on these proteins frequently have wide-ranging effects and are heterosubtypic. [31, 32] For example, it is known that a vaccine based on consensus sequence of the M2 protein ectodomain protects animals against influenza viruses of different strains, with either human or avian origins [14].

To create a vaccine that would provide effective immunization with respect to a wide range of different strains of influenza A virus from different hosts, we selected protein M2, an ionic channel, and protein NP as target antigens. The M2 antigen was chosen as a component that induces humoral immune responses, and NP antigen as a component that stimulates T-cell immune responses. We used the full-length sequence of M2 antigen, as the conserved antigenic epitopes are located not only in the ectodomain but also in the cytoplasmic domain, as recently shown by Muñoz-Medina J.E. et al. [20]. Moreover, such an approach eliminates the problem of low immunogenicity, as seen when using just the ectodomain. Challenges related to toxicity of the full-length M2 protein in eukaryotic cells were successfully overcome using an inducible promoter.

To obtain a vaccine with a wide-range of effects against influenza virus, highly conserved antigens are required because they contain the maximum quantity of conserved epitopes. However, according to bioinformatic analysis of sequences of highly conserved proteins of influenza virus, such as M2 and NP, their epitope content changes over time (within approximately the last 100 years). As a result, it becomes possible that a vaccine with a wide spectrum of effects created today may become ineffective in the near future. Therefore, we have created consensus sequences of M2 and NP enriched with epitopes.

To create an effective vaccine, one must choose an optimal approach to deliver specific antigens into the organism. It is possible to use genetic immunization that results in the delivery of target antigen genes directly into cells. Expression of genes that encode pathogen antigens imitates virus infection, and allows for the induction of high level humoral and cell-mediated responses. One of the most popular vectors for genetic immunization is recombinant human adenovirus, including human adenovirus serotype 5. These vectors are able to transduce different types of cells, including professional antigen-presenting cells, are suitable for intranasal injection, are able to activate innate immune responses and usually do not require the use of adjuvants. Replication-defective recombinant adenoviruses carry a deletion of the E1 genome area and are able to reproduce only in special packaging cell lines (HEK293, Per.C6). Candidate vaccine vectors based on serotype 5 recombinant human adenovirus have shown their effectiveness against different pathogens, including influenza A virus [33–36]. Furthermore, Tompkins et al. have shown that priming with a DNA-vaccine and boosting with a recombinant adenovirus that carries the gene for full-length M2 protein induces cross-humoral and cell-mediated responses, and also protects against lethal doses of H5N1 influenza virus [37]. A recombinant adenovirus that expresses the NP gene of influenza virus strain A/PR/8/34 (H1N1), when intranasally injected, can induce the production of specific IgA antibodies in murine respiratory tract mucosa [38]. Epstein et al. showed that priming with a DNA-vaccine and boosting with a recombinant adenovirus that expresses the NP gene induces the development of humoral and cell-mediated responses, and protects against influenza virus H5N1 [39]. Induction of protective immune responses is provided by immunization with a recombinant adenovirus that expresses the HA gene of avian influenza virus and the NP gene [40]. For this reason, to study obtained consensus sequences enriched for protein epitopes as antigens, we used a vector based on human adenovirus serotype 5.

By studying the immunogenicity of Ad5-tet-M2NP, it was shown that a single intranasal immunization in mice generates the production of antibodies specific to M2 and NP antigens. Similar levels of serum antibodies to M2 and NP antigens were observed after immunization of mice with doses of 10^7^ PFU Ad5-tet-M2NP. However, when a dose 10^8^ PFU Ad5-tet-M2NP was used, mice responded with more intensive production of antibodies to M2 in comparison with antibodies to NP (Fig 5A and 5B).

Using ELISPOT assays, we detected significant T-cell immune responses to M2 and NP antigens in mice that were immunized with Ad5-tet-M2NP (Fig 7A–7I). The ELISPOT method detects cells that secrete IFN-γ in response to reactivation of antigen-presenting dendritic cells. In the absence of adaptive immune responses, IFN-γ is secreted generally by NK or NKT cells. In the case of activation of the adaptive immune response, IFN-γ is generally produced by type 1 T-helper cells and cytotoxic T lymphocytes. IFN-γ functions in the activation of monocytes and macrophages. IFN-γ has great significance during the formation of Th1 and CD8 T cell immune responses [41]. Thus, the quantity of IFN-γ-producing cells may indicate the level of induction of T-cell immune responses.

Through the reactivation of splenocytes and sorted CD4+ or CD8+ T-cells by dendritic cells that were loaded with soluble NP or M2 antigens, we showed that, following immunization with Ad-tet-M2NP, murine IFN-γ-producing CD4+ T-cells were significantly produced (Fig 7G and 7H). In these conditions, more CD4+ T-cells reacted to NP epitopes than to M2 epitopes.

When dendritic cells that had been transduced with Ad5-tet-NP and Ad5-tet-M2 were used for reactivation of splenocytes, we revealed a significant quantity of IFN-γ-producing CD8+ T-cells that were able to recognize the corresponding NP or M2 epitopes (Fig 7D–7F). In this analysis, CD8+ T-cells sorted by ELISPOT reacted to NP epitopes (Fig 7F), but not M2 epitopes (Fig 7E). This difference in reactivity of CD8+ T-cells to M2 epitopes could be caused by the presence of antigen-reactive CD4+ helper T-cells together with CD8+ T-cells in the splenocyte culture.

The study of protective properties of Ad5-tet-M2NP showed that a single intranasal immunization with Ad5-tet-M2NP provides mice with protection against infection by a lethal dose of influenza A virus for different subtypes. To infect mice, we used influenza viruses of different phylogenetic groups that were obtained from different hosts (human, birds, swine). We have shown 100% protection of immunized mice against avian (H2N3, H5N2) and swine (H9N2) influenza viruses, and 90% protection against human influenza viruses (H1N1 and H3N2, Fig 11A–11E).

**Fig 12:**
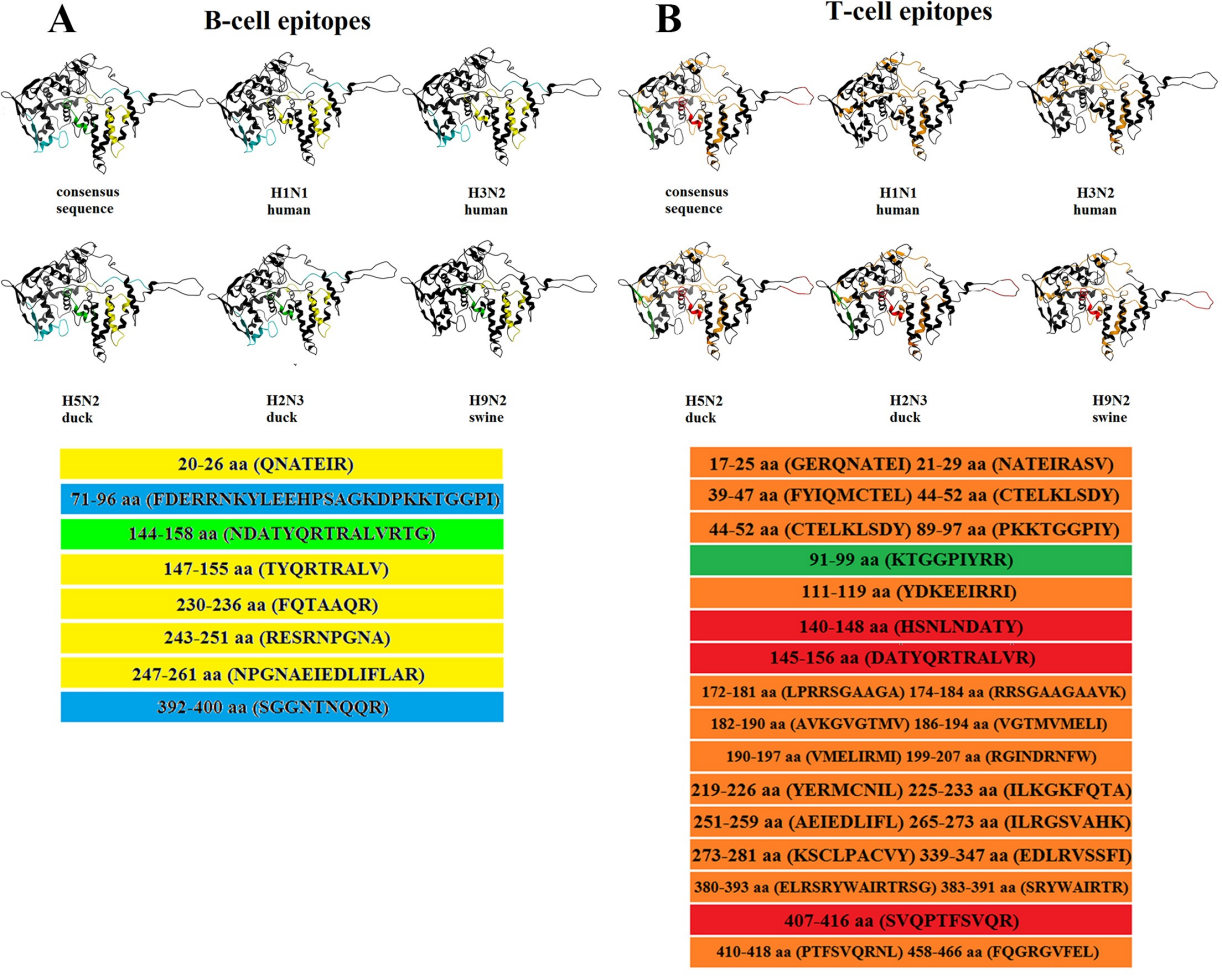
Occurrence of B and T-cell epitopes in the NP protein of influenza viruses that have different origins, and as a part of the consensus sequence. B (A) and T-cell epitopes (B) that occur in all the indicated viruses, are marked with yellow and orange colors, respectively; B-cell epitopes that occur in all viruses, except for those of swine origin, are marked with light-blue; B-cell epitopes that occur in all viruses, but are of human origin, are marked with light-green; T-cell epitopes that occur in all viruses, except for viruses of human origin, are marked with red; T-cell epitopes that occur in viruses originating from birds are marked with a dark-green color; 3D-structures of NP were plotted using the software ICM-Pro (http://www.molsoft.com/) on the basis of a structure obtained from Protein Data Bank (PDB:2Q06).

**Fig 13:**
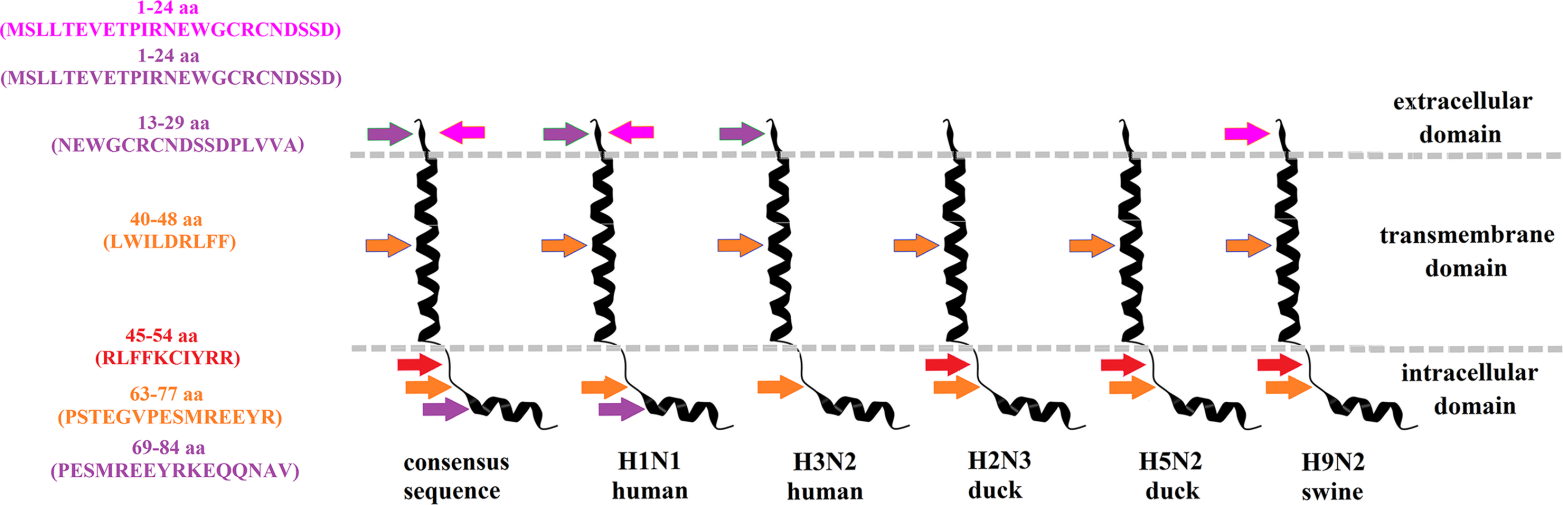
Occurrence of B and T-cell epitopes in the M2 protein of influenza viruses that have different origins, and as a part of the consensus sequence. T-cell epitopes that occur in all the indicated viruses are marked with orange; T-cell epitopes that occur in all viruses, except for viruses of human origin, are marked with red; T-cell epitopes that occur in viruses of human origin are marked with purple; B-cell epitopes that occur in human H1N1 and swine virus are marked in magenta.

High levels of protection in animals that were immunized with recombinant adenovirus, which expressed consensus sequences of NP and M2 proteins from influenza viruses that belong to different clades and originate from different hosts, may be explained by the fact that consensus-containing viral epitopes were obtained from 3 types of hosts. For instance, it was revealed that NP proteins hosted by birds, which we used for the infection of animals, contained 3 epitopes that were not present in the NP of human and swine viruses that we used for infection, but were present in the NP consensus sequence. A similar picture was observed with the M2 epitopes (Figs 12A, 12B and 13).

Protection against avian and swine influenza viruses was slightly better than against human influenza viruses. This may be due to a slightly higher content of T and B-cell epitopes that are 100% homologous with epitopes of bird and swine viruses (Table 2) in the NP consensus sequences.

**Table 2:** Content of T and B-cell epitopes that are 100% homologous with epitopes of human viruses in NP and M2 consensus sequences.

	NP			M2	
	T-cell epitopes		B-cell epitopes	T-cell epitopes	B-cell epitopes
	*Homo sapiens*	*Gallus gallus*			
**Consensus sequence**	**18/18**	**12/12**	**7/7**	**12/12**	**18/18**
**A/USSR/90/77 (H1N1)**	**14/18**	**9/12**	**7/7**	**10/12**	**18/18**
**A/Aichi/2/68 (H3N2)**	**14/18**	**10/12**	**7/7**	**9/12**	**18/18**
**A/Black Duck/New Jersey/1580/78 (H2N3)**	**18/18**	**12/12**	**7/7**	**3/12**	**0/18**
**A/Duck (Mallard)/Pensylvania/10218/84 (H5N1)**	**18/18**	**12/12**	**7/7**	**3/12**	**0/18**
**A/Swine/Hong Kong/9A-1/98 (H9N2)**	**17/18**	**8/12**	**5/7**	**3/12**	**0/18**

Thus, a single intranasal immunization of Ad5-tet-M2NP is able to induce protective immune responses against a broad-spectrum of influenza A virus subtypes that belong to 3 different clades and originate from 3 different hosts (Table 1). However, we should note that Ad5-tet-M2NP preparation generally neutralizes influenza virus after its penetration into the cell. In the future, we would likely need to include a component that neutralizes influenza virus outside of the cell. This component can be developed on the basis of conserved epitopes of hemagglutinin, particularly its stalk-domain. Thus, our obtained data allow us to envision using Ad5-tet-M2NP as a basis for the development of a universal vaccine against influenza A viruses.

## Materials and methods

### Reagents

The following reagents were used in this work: Ad-Easy vector system (Stratagene, USA); bovine serum albumin (BSA), mouse monoclonal anti-M2 (14С2) IgG (Abcam, United Kingdom); mouse monoclonal anti-NP (В248М) IgG (Abcam, United Kingdom); Donkey Anti-Goat IgG H&L (DyLight® 488) (Abcam, United Kingdom), HRP-conjugated sheep anti-mouse IgG (Amersham Bioscience, USA), recombinant influenza A virus A/Puerto Rico/8/34/Mount Sinai (H1N1) NP (Sinobiological, China) and chemically synthesized M2 ectodomain (MSLLTEVETPIRNEWGCRCNDSSD) (Almabion, Russia).

### Cell lines

Human embryonic kidney-293 (HEK-293) (Russian collection of vertebrate cell lines, Saint Petersburg, Russia) and adenocarcinomic human alveolar basal epithelial (A549) (Russian collection of vertebrate cell lines, Saint Petersburg, Russia) cells were used in the experiments. Cells were propagated in DMEM (GE Healthcare, USA) supplemented with 10% fetal bovine serum (HyClone), 100 U/ml penicillin, 100 mg/ml streptomycin, and 2 mM L-glutamine (all PanEco, Russia) at 37° C in a humidified atmosphere with 5% CO_2_.

### Viruses and bacterial strains

The following mouse-adapted viruses were used in this work: A/USSR/90/1977(H1N1) (GenBank: KX879554.1, KX879555.1, KX879556.1, KX879560.1, KX879561.1, KX879557.1, KX879558.1, KX879559.1), A/black duck/New Jersey/1580/1978(H2N3) (GenBank: KX879562.1, KX879563.1, KX879564.1, KX879566.1, KX879567.1, KX879568.1, KX879569.1, KX879565.1), A/duck/Pennsylvania/10218-MA/1984(H5N2) (GenBank: KX879585.1, KX879584.1, KX879583.1, KX879582.1, KX879581.1, KX879580.1, KX879579.1, KX879578.1), A/swine/Hong Kong/9/98(H9N2) (GenBank: KX879593.1, KX879592.1, KX879591.1, KX879590.1, KX879589.1, KX879588.1, KX879587.1, KX879586.1), A/Alchi/2/1968(H3N2) (GenBank: KX879577.1, KX879576.1, KX879575.1, KX879574.1, KX879573.1, KX879572.1, KX879571.1, KX879570.1) (State collection of viruses. Federal Research Centre of Epidemiology and Microbiology named after Honorary Academician N. F. Gamaleya, Ministry of Health, Moscow, Russia) [28].

Escherichia coli DH5α (Invitrogen, USA) and BJ5183 (Stratagene, USA) strains were used.

### Development of optimal amino acid sequences of M2 and NP antigens

Original sequences of the M2 and NP proteins from different human strains of influenza A virus were retrieved from the international Influenza Virus Database (http://www.ncbi.nlm.nih.gov/genomes/FLU/Database/nph-select.cgi?go=database). We used all full-length sequences of NP and M2 from influenza viruses obtained from humans prior to 2014, but we excluded identical sequences. The sequences were aligned relative to each other, and their consensus sequences were compiled in silico using Geneious 4.8.5 software. To enrich obtained consensus sequences with conserved and evolutionarily significant T and B-cell epitopes, it was necessary to choose such epitopes. We have browsed the web-site IEDB (Immune Epitope Database and Analysis Recourse, http://www.iedb.org/) for all experimentally confirmed T and B-cell epitopes of human, avian and swine influenza virus NP and M2 proteins. Then, we divided all sequences of NP or M2 for influenza viruses from which consensus sequences were obtained into sets of approximately 100 sequences, in accordance with their chronological collection. The number of sequences in each set was restricted by the completeness of the time period, for example a whole year, in which the corresponding viruses were collected. Sequences of T and B-cell epitopes were then aligned with sets of sequences of NP or M2. Alignment was performed with the assistance of the service IEDB (http://www.iedb.org/ - Epitope Conservancy Analysis). Epitopes completely matching more than 50% of sequences from one time period at least twice in history (two periods that are separated from each other by one time period in which an epitope matching less than 50% of sequences) were included in consensus sequences (S1 Table). Eventually, we found consensus sequences of M2 and NP that have the maximum quantity of epitopes that are 100% coincident with them.

To enhance the NP protein immunogenicity, its sequence was modified by the removal of an unconventional nuclear localization signal. For this, a T6KR8 motif was replaced by an A6AA8 motif, according to Ohba K. et al. [42].

### Design of genetic constructs expressing universal consensus sequences of M2 and NP proteins

For the next step of our work, consensus epitope-enriched amino acid sequences of M2 and NP proteins were converted into nucleotide sequences and linked together through a nucleotide sequence (found in UNIPROT [UniProtKB/Swiss-Prot P03306] database) that encode the self-cleaving 2A peptide (QLLNFDLLKLAGDVESNPGP) of foot-and-mouth disease virus (strain A10-61) (Aphthovirus A). Genetic constructs encoding M2 and NP protein consensus amino acid sequences and the 2A peptide nucleotide sequence were designed in silico using the Geneious 4.8.5 software package. Codons of M2 and NP genes were adapted for expression in mammalian cells according to the database of coding triplets - http://www.kazusa.or.jp/codon/. The nucleotide sequences encoding M2 and NP were obtained using chemical synthesis by Evrogen JSC and cloned into plasmid pAL-TA (pAL-TA-M2-NP) ourselves or by Evrogen.

### Construction of recombinant human adenoviruses expressing the consensus sequences of M2 and NP proteins

Plasmid pShuttle-tet-off-tTA, carrying regulatory elements of the tet-off system, was obtained by cloning the required areas from plasmids pTet-off and pTRE-Tight (Tet-Off & Tet-On Gene Expression System, Clontech, USA) into plasmid pShuttle-CMV, using KpnI and EcoRV restriction endonucleases.

M2, NP and M2-2A-NP nucleotide sequences were cloned into the pShuttle-Tet-off-tTA plasmid. As a result, we have 3 plasmid structures: pShuttle-Tet-off-M2, pShuttle-Tet-off-NP and pShuttle-Tet-off-M2NP. Therefore, a genetic construct that carry M2 and NP gene nucleotide sequences, which are linked by the 2A peptide coding nucleotide sequence under control of the Tet-off expression system, was developed (Fig 3A).

The Ad-Easy Adenoviral Vector System was used to construct Ad5-tet-M2, Ad5-tet-NP and Ad-tet-M2NP according to the manufacturer’s instructions. The rAd with the E1 region replaced with a transgene-free expression cassette (Ad-null) was used as a control. rAds were grown in HEK-293 cells and chromatographically purified. The titers of Ad5-tet-M2, Ad5-tet-NP, Ad-tet-M2NP and Ad5- null (5 x 10^9^ PFU/ml, 7 x 10^9^ PFU/ml, 5 x 10^9^ PFU/ml and 3 x 10^9^ PFU/ml, respectively) were determined by plaque formation assay in HEK-293 cell culture [43].

### Detection of M2 and NP protein gene expression as a part of Ad5-tet-M2NP

To detect expression of the M2 and NP genes, we used immunohistochemistry. A549 cells were plated on plates with germfree cover-glass for confocal microscopy. After 18 hours, cells were transduced with Ad5-tet-M2NP. 24 hours following transduction, the cells were rinsed with PBS solution and then were fixed with a 70% acetone solution. After fixation, they were washed three times with distilled water and three times with PBS and 0.05% Tween 20 (TPBS) solution. Then, we incubated the cells at 37°C for 30 minutes on a shaker with buffer containing 5% non-fat dried milk in TPBS, then washed them again as described above and incubated the cells at 37°C for 1 hour on the shaker with anti-NP or anti-M2 primary antibodies (1:500), followed by a 1:500 dilution of goat anti-mouse IgG conjugated with the DyLight® 488 (1:500) fluorescent tag as a secondary antibody. In the closing stage of the study, we washed the cells from the conjugate and treated them with DAPI-staining solution for 1 minute at room temperature. Visualization of fluorescent cells was achieved by confocal microscopy, using a Nikon Confocal microscope C-1 and EZ-C1 software ver. 3.90.

### Immunization and challenge infection of mice

Six-week old female Balb/c mice were obtained from the Pushchino Branch of the Institute of Bioorganic Chemistry, RAS (Pushchino, Russia). The mice had free access to water and standard rodent chow and were housed in pathogen-free cages. Intranasal (i.n.) administration (rAds) and challenge infections were performed as described [44].

For immunization, mice weighing 18-20g were treated with two different doses of Ad5-tet-M2NP (10^7^ and 10^8^ PFU/mouse for low- and high-dose immunizations, respectively), Ad5-null as a negative control (10^8^ PFU/mouse) or PBS. For the infection challenge, 30 days after immunization, animals were intranasally infected with 10x LD50 of influenza A viruses in a total volume of 0.05 ml. LD50 for each of the viruses used in the study are in the Table 3.

**Table 3:** Titers of influenza viruses.

IAV	Titer, LD50/ml
H1N1 (A/USSR/90/77)	2000
H2N3 (A/BlackDuck/NewJersey/1580/78)	4000
H5N2 (A/Duck(Mallard)/Pennsylvania/10218/84)	80000
H9N2 (A/Swine/Hong Kong/9A-1/98)	10000
H3N2 (A/Aichi/2/68)	50000

After challenge, mice were monitored twice daily for mortality and morbidity (body weight, clinical score of influenza infection) checks for a 14-day observation period, until humane endpoint or found dead. Humane endpoint was based on clinical score.

We used 4-point clinical scoring system to determine the indications for euthanasia [45, 46]. Institutional Animal Care and Use Committee (IACUC) of Federal Research Centre of Epidemiology and Microbiology named after Honorary Academician N.F. Gamaleya agreed to allow clinical score 4 as a humane endpoint. The clinical scores for challenges were defined as: 0 = no clinical signs, 1 = rough coat, 2 = rough coat, less reactive, passive during handling, 3 = rough coat, rolled up, labored breathing, passive during handling, 4 = rough coat, rolled up, labored breathing, unresponsive (= humane endpoint). Mice that reached humane endpoint were sacrificed by the use of CO2 for rodent euthanasia with following cervical dislocation for death confirmation. Once animals reached endpoint criteria they were immediately sacrificed. 25 from 44 of animals that were died from influenza infection were found dead before meeting criteria for euthanasia. All other mice were monitored twice daily for 14 days and were euthanized at the end of experiments (at day 15).

### Ethics statement

All of the experimental procedures conform to the Guide for the Care and Use of Laboratory Animals published by the National Institutes of Health (NIH Publication #85–23, revised 1996) and National Standard of the Russian Federation GOST R 53434–2009, approved by Institutional Animal Care and Use Committee (IACUC) of Federal Research Centre of Epidemiology and Microbiology named after Honorary Academician N.F. Gamaleya and were performed under Protocols #Imb-2016-033, #Imb-2016-034, #Imb-2016-056, #Imb-2016-057. All persons using or caring for animals in research underwent yearly training as required by the IACUC.

### Study of humoral immune responses

We took blood samples from mice from the vena caudalis. The samples were incubated at 37°C for 30 minutes until clotting occurred. Then, the serum was transferred to another tube and was centrifuged at 3000 rpm for 10 minutes. 400 μL aliquots of this serum were distributed to tubes and stored at -20°C.

To determine IgG titers to influenza virus M2 and NP proteins in the serum of immunized mice, we used an indirect enzyme-linked immunosorbent assay (ELISA). We investigated individual samples of sera of 10 mice from each experimental group. Recombinant NP protein or chemically synthesized M2 protein ectodomain were adsorbed at the immunoplate at concentrations of 5 μg/μL. Next steps of ELISA have been carried out as it was described earlier [47, 48].

The level of serum antibodies for proteins from influenza viruses of different subtypes was evaluated as described above. The only difference was that in the wells of the plate for ELISA, we placed the lysate of one of the following subtypes of influenza virus: H1N1 (A/USSR/90/77), H2N3 (A/BlackDuck/NewJersey/1580/78), H5N2 (A/Duck(Mallard)/Pennsylvania/10218/84), H9N2 (A/Swine/Hong Kong/9A-1/98) or H3N2 (A/Aichi/2/68).

### T-cell immune responses to antigenic epitopes of M2 and NP proteins

The quantity and phenotype of T-cells that react to antigens M2, NP and Ad5 were detected using ELISPOT and flow cytometry with a suspension of mononuclear cells, and in refined fractions of immunized mice splenic CD4 and CD8 T-cells of immunized mice. T-cells were reactivated in vitro in co-cultures with antigen-presenting dendritic cells derived from syngeneic unimmunized mice. T-cells were reactivated in vitro in cocultures with antigen-presenting dendritic cells.

To obtain cell-rich fluid, mice were sacrificed via CO2 inhalation with following cervical dislocation for death confirmation. The spleen and bone marrow were extracted in aseptic conditions as described by Madaan A. et al [49]. Cell-rich splenic fluid was sorted by ficoll (density 1.09 g/cm3) to obtain mononuclear cell preparations [50].

For restimulation of CD4 T-cells in vitro, we used dendritic cells that present antigenic M2 or NP epitopes within the context of MHC class II. For this purpose, dendritic cells were pre-activated by lipopolysaccharide E. coli 055:B5 (1 μg/ml) and were loaded with recombinant protein NP (5 μg/ml) or M2e peptide (50 μg/ml). We also used dendritic cells that were not loaded with any influenza virus antigens as negative controls. During studies on cross-reactions of T-cells to antigens of different influenza A virus subtypes, dendritic cells were incubated with lysates of inactivated viruses H1N1 (A/USSR/90/77); H2N3 (A/BlackDuck/NewJersey/1580/78), H3N2 (A/Aichi/2/68), H5N2 (A/Duck(Mallard)/Pennsylvania/10218/84) or H9N2 (A/Swine/Hong Kong/9A-1/98) with 10 μg/ml concentrations of protein. For preparation of virus lysates the viruses had been inactivated by UV radiation and ultrasonication. Protein concentration in lysates was determined with a BCA kit (Protein assay reagent, Pierce).

For in vitro restimulation of CD8 T-cells, we used dendritic cells that present antigenic epitopes of M2, NP or of adenovirus within the context of MHC class I. In this instance, dendritic cells were transduced with Ad5-tet-M2, Ad5-tet-NP or Ad5-null (100 PFU per cell), correspondingly.

### Detection of IFN-y-secreting T-cells by ELISPOT

Detail description of this method see in protocols.io: http://dx.doi.org/10.17504/protocols.io.jn4cmgw. 96-well plates for ELISPOT (mouse IFN-γ ELISPOT Kit, cat. 552569, BD Biosciences) were seeded with 10^4^ dendritic cells in 150 μl of complete culture medium per well. Antigen-presenting dendritic cells were obtained as described above. After dendritic cells were added to the wells of the plates, 50 μl of the splenic cell suspension of interest– 5x10^5^ mononuclear splenic cells or 10^5^ graded CD4+ T-cells or 5x10^4^ graded CD8+ T-cells for each well—were added. The plate was incubated for 20 hours in a CO_2_-incubator strictly in the horizontal position, avoiding shaking. Then, the plate was treated in accordance with manufacturer’s protocols (BD Biosciences). Wells of the plates were washed several times with distilled water and then 3 times with wash buffer (BD Biosciences). We added to each well 100 μl of detecting antibodies for IFN-y and incubated them for 2 hours at room temperature. Then, we washed them 4 times with wash buffer and added 100 μl/well of streptavidin-peroxidase conjugate (BD Biosciences), and incubated them for 1 hour. After the incubation, we elaborately washed wells with wash buffer and then PBS solution. We quickly added chromogenic substrate of 3-amino-9-ethylcarbazole (BD Biosciences), incubated them for 15 minutes and after that elaborately washed them with distilled water.

We imaged each well after drying at room temperature using a binocular microscope MBS-10 (magnification x4) and digital camera (Levenhuk DCM800 with 1280x960 pixel resolution). Quantification of spots with IFN-y that had been formed by single cells in the wells was performed by using the software package Mathematica 8.0 (Wolfram Research).

### Determination of T-cells that synthesize IFN-γ by flow cytofluorometry

Detail description of this method see in protocols.io: http://dx.doi.org/10.17504/protocols.io.jn4cmgw. 10^5^ dendritic cells (reference ones or transduced Ad5-tet-M2 or Ad5 tet-NP) in 1 ml of complete culture medium were added to each well of a 24-well Nunclon culture plate, followed by 5 million mononuclear splenic cells in 100 μl of PBS-cell. The plate was incubated for 12 hours in a CO2-incubator, then brefeldin-A at a final concentration of 5 μM was added. After 6 hours of incubation, 2 ml of cold PBS-cyto (PBS without magnesium and calcium with the addition of 10 mM HEPES, 0.5% BSA, 0.01% sodium azide, 0.35 mM EDTA, pH 7.4) was added, and cells were carefully pipetted and transferred to 4.5 mm-test-tubes (BD Biosciences). Then, cells were pelleted (10 minutes, 1200 rpm), the cell pellet was mixed with 1 ml of PBS-cyto and transferred to microtubes of 1.2 ml volume.

The cells were centrifuged once more (10 minutes, 1200 rpm) and a mixture of antibodies (CD4/ CD8/ CD44/ CD62L) was added to the cell pellet. After 20 minutes of dark incubation at 4°C, the cell pellet was washed with PBS-cyto. The cell pellet was then stirred, and 100 μl of FixPerm solution was added. After 20 minutes of incubation, the cell pellet was washed with PermWash solution two times. The cell pellet was resuspended in 50 μl of PermWash and divided into 2 aliquots (20 μl each), and then IFN-γ antibodies were added. It was incubated for 30 minutes, washed with PermWash solution two times, resuspended in 200 μl of PBS-cyto and analyzed with a flow cytofluorometer FAC AriaII. The samples of interest were analyzed in duplicate. The data are presented as average values and standard deviations.

### Statistical analysis

Statistical data was analyzed using Statistics software (StatSoft). For quantification of interferon-γ secreting T cells was used Mann–Whitney–Wilcoxon nonparametric test. For antibody titers and infection study The Mann–Whitney U test was used to evaluate differences in antibody titers in ELISA. Changes in body weights and survival were measured by calculating percentage compared with baseline in each group. Comparison of survival used Mann-Whitney test and Gehan-Wilcoxon test. p<0.05 indicates statistical significance.

## Supporting information

S1 Table Results of alignment of T and B-cell epitopes with M2 and NP consensus sequences, challenge viruses and influenza viruses obtained from humans before 2014 with hemagglutinin type H1, H2, H3 and neuraminidase type N1 and N2, and from human H5N1 viruses. Epitopes that are completely contained in NP and M2 of challenge viruses and epitope-enriched sequences are marked with color filling. B and T-cell epitopes that occur in the indicated viruses are marked with yellow and orange colors, respectively; B-cell epitopes that occur in all viruses, except for swine ones, are marked with light-blue; B-cell epitopes that occur in all viruses, but are of human origin, are marked with light-green; T-cell epitopes, that occur in all viruses, except for the viruses of human origin, are marked with red; T-cell epitopes that occur in viruses originating from birds are marked with the dark-green color; T-cell epitopes that occur in all viruses, but are of human origin, are marked with purple; B-cell epitopes that occur in human H1N1 and swine virus are marked with magenta.
(XLSX)
